# Supplementary material for: Systematic media review: A novel method to assess mass-trauma epidemiology in absence of databases—A pilot-study in Rwanda
Source: PLoS One. 2021 Oct 13;16(10):e0258446. doi: 10.1371/journal.pone.0258446 (PMC8513851; doi:10.1371/journal.pone.0258446)
Supplement: S1 Appendix — (DOCX) [file pone.0258446.s001.docx]

**Appendix 1.** NexisUni Search strategy

Date Range: Jan 1, 2010 – September 1, 2020

*ENGLISH*

hlead(Rwanda) and hlead((accident*)) and hlead((road* OR traffic* OR vehicle* OR car* OR moto* OR motorcycle* OR bus* OR truck*)) and hlead((dead OR death* OR fatal* OR injur* OR wound* OR trauma OR kill*))

398 unique articles

hlead(Rwanda) and hlead((flood* OR landslide* OR earthquake* OR cyclone* OR storm* OR hurricane* OR natural hazard* OR natural disaster*)) and hlead((dead OR death* OR fatal* OR injur* OR wound* OR trauma OR kill*))

707 unique articles

hlead(Rwanda) and hlead((shoot* OR machete* OR stab* OR explosion* OR detonation* OR weapon*) ) and hlead((dead OR death* OR fatal* OR injur* OR wound* OR trauma OR kill*))

1,921 unique articles

**Total number of articles:** 3,026

*FRENCH*

hlead(Rwanda )AND hlead((accident* de la circulation OR accident* de la route OR accident* de moto* OR accident* de bus OR accident* de voiture OR accident* automobile* OR accident* de camion*)) AND hlead((blessure* OR blesse* OR traumatisme* OR mort*))

23 unique articles

hlead(Rwanda) AND hlead((glissement de terrain OR eboulement* OR inundation* OR tremblement de terre OR seisme* OR cyclone* OR ouragan* OR tempete* OR catastrophe* naturelle* OR désastre*)) AND hlead((blessure* OR blesse* OR traumatisme* OR mort*))

31 unique articles

hlead(Rwanda) AND hlead((blesse* par balle OR arme* à feu* OR arme* blanche* OR machette* OR explosion* OR detonation*)) AND hlead((blessure* OR blesse* OR traumatisme* OR mort*))

107 unique articles

**Total number of articles:** 161

*KINYARWANDA*

hlead(Rwanda ) and hlead(impanuka) and hlead((impanuka zo mumuhanda OR imodoka OR moto OR bus OR amakamyo)) and hlead((urupfu OR ibikomere bitera urupfu OR impanuka OR gupfa))

0 unique articles

Rwanda AND (imyuzure OR isuri OR imitingito OR serwakira OR inkubi y’umuyaga OR Ibiza) AND (urupfu OR ibikomere bitera urupfu OR impanuka OR gupfa)

0 unique articles

Rwanda AND (kurasa OR umupanga OR gusogota OR guturika OR intwaro) AND (urupfu OR ibikomere bitera urupfu OR impanuka OR gupfa)

0 unique articles
