# Supplementary material for: Systematic media review: A novel method to assess mass-trauma epidemiology in absence of databases—A pilot-study in Rwanda
Source: PLoS One. 2021 Oct 13;16(10):e0258446. doi: 10.1371/journal.pone.0258446 (PMC8513851; doi:10.1371/journal.pone.0258446)
Supplement: S2 Appendix — (DOCX) [file pone.0258446.s002.docx]

**Appendix 2.** REDCap Data Extraction Form

- **Article title:** __________
- **Who published article?** (newspaper name) __________
- **Language of article:** English / French / Kinyarwanda / Other
- **Date of publication (YYMMDD):** __________
- **Date of trauma event (YYMMDD):** __________
- **Geographical location of the event (province):** Northern / Western / Southern / Eastern / Kigali / Not mentioned or other
- **In which district did it occur?** Gasabo (Kigali) / Kicurkiro (Kigali) / Nyarugenge (Kigali) / Burera (Northern) / Gakenke (Northern) / Gicumbi (Northern) / Musanze (Northern) / Rulindo (Northern) / Gisagara (Southern) / Huye (Southern) / Kamonyi (Southern) / Muhanga (Southern) / Nyamagabe (Southern) / Nyanza (Southern) / Nyaruguru (Southern) / Ruhango (Southern) / Bugesera (Eastern) / Gatsibo (Eastern) / Kayonza (Eastern) / Kirehe (Eastern) / Ngoma (Eastern) / Nyagatare (Eastern) / Rwamagana (Eastern) / Karongi (Western) / Ngororero (Western) / Nyabihu (Western) / Nyamasheke (Western) / Rubavu (Western) / Rusizi (Western) / Rutsiro (Western) / Other or unknown
- **What was the mechanism of trauma?** Road traffic accident / natural hazards / acts of violence/terroris / other
  - If “road traffic accident”:
    - **What type of vehicles/persons were involved in the road traffic accident?** Bus / motorcycle / car / truck / bicycle / pedestrians / other
    - **What happened in the accident?** Collision / derailment (slid off the road) / Other
  - If “natural hazard”:
    - **What type of natural hazard was it?** Landslide or flooding / earthquake / storm, hurricane or cyclone / other
  - If “acts of violence/terrorism”:
    - **What type of acts of violence/terrorism-related mechanism** **was it?** Machete or knife / gun, rifle or firearm / explosion or detonation / other
- **Number of persons injured (not dead):** __________
- **Number of on-site deaths:** __________
- **Any other relevant information shared:** __________
