# Supplementary material for: Systematic media review: A novel method to assess mass-trauma epidemiology in absence of databases—A pilot-study in Rwanda
Source: PLoS One. 2021 Oct 13;16(10):e0258446. doi: 10.1371/journal.pone.0258446 (PMC8513851; doi:10.1371/journal.pone.0258446)
Supplement: S3 Appendix — (DOCX) [file pone.0258446.s003.docx]

**Appendix 3.** Included articles

| Article title | Media source | Year of publication |
| --- | --- | --- |
| Focus On Africa - 5:30 PM GMT | TVeyes - BBC World | 2019 |
| Rwanda; Two Children Die in Grenade Explosion | The New Times (Kigali) | 2010 |
| Rwanda; Accident Claims Three Road Workers | The New Times (Kigali) | 2010 |
| Rwanda; One Dead, 24 Injured in Road Accident | The New Times (Kigali) | 2010 |
| Grenade blast wounds 16 in Rwandan capital: police | Agence France Press | 2010 |
| Grenade explodes in western Rwanda as two other attacks hit capital 4 | BBC Monitoring Africa - Political | 2010 |
| Un mort et 16 blessés dans une série d'eplosions à Kigali | Xinhua News Agency - French | 2010 |
| Rwanda: Floods kill four in western district of Rubavu | BBC | 2010 |
| Rwanda: Floods Kills Four in Western Province | The New Times | 2010 |
| Rwanda: Twenty feared dead as boat capsizes in Lake Kivu | BBC | 2010 |
| Rwanda; 20 Mourners Feared Dead as Boat Capsizes in L. Kivu | The New Times | 2010 |
| Rwanda; Five Seriously Injured in Kanogo Accident | The New Times (Kigali) | 2010 |
| Rwanda; 40 Injured in Two Separate Road Accidents | The New Times (Kigali) | 2010 |
| Rwanda; 40 Injured in Two Separate Road Accidents | The New Times (Kigali) | 2010 |
| Rwanda; 12 Injured in Road Accident | The New Times (Kigali) | 2010 |
| Rwanda; 12 Injured in Road Accident | The New Times (Kigali) | 2010 |
| Pair of grenade attacks kills 2 and hurts 32 in Rwanda; Explosions rock capital as country prepares for presidential election | The International Herald Tribune | 2010 |
| Rwanda; One Dead, 28 Wounded in Grenade Attacks | The New Times (Kigali) | 2010 |
| Rwanda Blast Critically Wounds 7 | SmarTrend | 2010 |
| Rwanda; Grenade Victim Dies | The New Times (Kigali) | 2010 |
| Rwanda: explosion d'une grenade à Kigali, au moins sept blessés (police) | Agence France Press | 2010 |
| Rwanda: explosion d'une grenade à Kigali, au moins sept blessés (police) | La Tribune de Geneve.fr | 2010 |
| Rwanda : explsion d'une grenade à Kigali (presse) | Xinhua News Agency - French | 2010 |
| Point de l'actualité à 21H00 GMT | Xinhua News Agency - French | 2010 |
| Rwanda grenade attack marks Kagame's presidential win | Agence France Press - English | 2010 |
| AF-Rwanda-Election, Update | The Canadian Press | 2010 |
| Rwanda vote terror | The Advertiser (Australia) | 2010 |
| Rwanda; 25 Injured in Road Accidents in Rwamagana District | The New Times (Kigali) | 2010 |
| Rwanda; Seven Injured in City Road Accident | The New Times (Kigali) | 2010 |
| Rwanda; Three Dead, 13 Injured in Road Accident | The New Times (Kigali) | 2010 |
| Rwanda: Six Miners Killed by Landslide | The New Times | 2010 |
| wanda; Canadian Family Survives Fatal Accident | The New Times (Kigali) | 2011 |
| Seven injured in grenade explosion in Rwanda | BBC Monitoring Africa - Political | 2011 |
| Two killed in Rwanda grenade explosion | Agence France Press | 2011 |
| Two killed, 26 injured in grenade explosion in Rwandan capital | BBC Monitoring Africa - Political | 2011 |
| Rwanda: deux morts et 26 blessés dans l'explosion d'une grenade à Kigali | Agence France Press | 2011 |
| L'explosion d'une grenade fait deux morts près de Kigali, au Rwanda | The Canadian Press | 2011 |
| Rwanda; One Dies in Bus Accident | The New Times (Kigali) | 2011 |
| 10 injured in grenade explosion in Rwanda | Xinhua General News Service | 2011 |
| Rwanda : une explosion à la grenade fait 10 blessés | Xinhua News Agency - French | 2011 |
| 5 people dead in Rwanda boat collision | Xinhua General News Service | 2011 |
| Rwanda; Over 30 Injured in Bus Accident | The New Times (Kigali) | 2011 |
| Rwanda; 11 Perish in Road Accident | The New Times (Kigali) | 2011 |
| Rwanda; Five Injured in Accident | The New Times (Kigali) | 2011 |
| Grenade explosion injures 21 in western Rwanda | BBC Monitoring Africa - Political | 2011 |
| Rwanda; Grenade Blast Injures 21 in Rusizi | The New Times (Kigali) | 2011 |
| Grenade blast wounds 21 in Rwanda | Agence France Press | 2011 |
| Au Rwanda, plusieurs dizaines de blessés après un attentat à la grenade... | Renseignor | 2011 |
| Rwanda; 15 Injured in Commuter Taxi Accident | The New Times (Kigali) | 2011 |
| Rwanda; Landslide in Nyabihu Kills Three Teenagers | The New Times | 2011 |
| Rwanda; Five Injured in Accident | The New Times (Kigali) | 2011 |
| Rwanda: Lighting kills student as floods destroy 12 homes | The New Times | 2011 |
| Rwanda; Inatek Teacher Killed in Car Accident | The New Times (Kigali) | 2011 |
| Rwanda: 10 Killed in Karongi Floods | The New Times | 2011 |
| Rwanda; Children Who Perished in Landslide Laid to Rest | The New Times | 2011 |
| Rwanda; Two Killed in Rulindo Accident | The New Times (Kigali) | 2011 |
| Rwanda; Grenade Explosion Kills Two | The New Times (Kigali) | 2012 |
| Rwanda: deux morts dans l'explosion d'une grenade à Kigali (police) | Agence France Press | 2012 |
| Nouvelle attaque à la grenade à Kigali | Radio France Internationale | 2012 |
| Grenade blast in Rwanda was criminal act: police | Agence France Press | 2012 |
| Dix blessés dans l'explosion d'une grenade dans le centre du Rwanda | Agence France Press | 2012 |
| Rwanda; Three Survive Fatal Road Accident | The New Times (Kigali) | 2012 |
| Rwanda; Six Injured in Road Accident | The New Times (Kigali) | 2012 |
| Rwanda; Eight Killed in Bus Accident | The New Times (Kigali) | 2012 |
| Explosion in Rwanda leaves one dead, five injured:police | Agence France Press | 2012 |
| Rwanda: un mort et 5 blessés dans une explosion dans le nord (police) | Agence France Press | 2012 |
| Rwanda: six blessés par deux grenades dans Kigali et sa banlieue (police) | Agence France Press | 2012 |
| Rwanda: Flood Death Toll Rises | The New Times | 2012 |
| Rwanda; Floods Kill Two in Musanze, Nyabihu | The New Times | 2012 |
| Rwanda floods kill 8 people | Xinhua General News Service | 2012 |
| In Brief: Floods displace hundreds in Rwanda | IRIN Africa Service | 2012 |
| Rwanda; Floods Kill, Displace Many in Nation | East African Business Week | 2012 |
| Rwanda; Two Killed, 11 Injured in Road Accident | The New Times (Kigali) | 2012 |
| Rwanda; Truck Rams Into House, Kills 8 | The New Times (Kigali) | 2012 |
| Rwanda; Eight Killed in Fatal Car Accident | The New Times (Kigali) | 2012 |
| Rwanda; Five Perish in Rubavu Road Accident | The New Times (Kigali) | 2012 |
| Rwanda; Heavy Rains Kill One, Destroy Property | The New Times | 2012 |
| Rwanda; One Dead, Six Injured in City Accident | The New Times (Kigali) | 2012 |
| Rwanda; Pupil Killed in Road Accident | The New Times (Kigali) | 2012 |
| Rwanda; Kamonyi Accident Claims Two | The New Times (Kigali) | 2012 |
| Rwanda; Four Policemen Killed in Nyanza Road Accident | The New Times (Kigali) | 2013 |
| Rwanda; Nyanza Accident Kills Four Policemen | The New Times (Kigali) | 2013 |
| Rwanda; Musanze Accident Claims One, Injures Six | The New Times (Kigali) | 2013 |
| Rwanda; Gloomy Trip. Nur Students Injured in Road Accident | The New Times (Kigali) | 2013 |
| Rwanda; Nur Students Injured in Road Accident | The New Times (Kigali) | 2013 |
| Nur Students Injured in Road Accident | The New Times (Kigali) | 2013 |
| Rwanda; Grenade Blast Kills One | The New Times (Kigali) | 2013 |
| Rwanda; Two Arrested Over Grenade Explosion | The New Times (Kigali) | 2013 |
| Rwanda: Un tué, huit blessés dans un attentat à la grenade à Kigali (police) | Agence France Press | 2013 |
| Rwanda - Rwanda : attentat meurtrier à Kigali | RFI | 2013 |
| Rwanda: attentat meurtrier à Kigali | Le Figaro Newsflash | 2013 |
| Rwanda; Six Killed, Several Injured in Butare Road Accident | The New Times (Kigali) | 2013 |
| Rwanda; Six Die in Huye Road Accident | The New Times (Kigali) | 2013 |
| Rwanda; 50 Survive Road Accident | The New Times (Kigali) | 2013 |
| Rwanda; Six Dead, 16 Injured in Kirehe Road Accident | The New Times (Kigali) | 2013 |
| Rwanda; Six Dead, 16 Injured in Kirehe Road Accident | The New Times (Kigali) | 2013 |
| Rwanda; Six People Killed in a Accident | The New Times (Kigali) | 2013 |
| Three arrested following fatal grenade attack | Economist Intelligence Unit (EIU) Country Reports | 2013 |
| Grenade blast in Rwanda capital kills 2 people, leaves others seriously wounded, police say; Police: 2 people dead in grenade blast in Rwanda | Canadian Press | 2013 |
| Police: 2 people dead in grenade blast in Rwand | The New Zealand Herald | 2013 |
| Rwanda; Two Dead, Eight Critically Injured in Rusizi Accident | The New Times (Kigali) | 2013 |
| Rwanda; Two Die, Eight Hurt in Rusizi Road Accident | The New Times (Kigali) | 2013 |
| Rwanda; Heavy Rains Kill Two Children in Nyagatare | The New Times | 2013 |
| Explosive Start to Rwandan Parliamentary Election Polling Mars Kagame's Victory | ThinkAfricaPress | 2013 |
| Rwanda: deux personnes tuées par des grenades avant les législatives (police) | Agence France Press | 2013 |
| Rwanda : La campagne pour les législatives perturbée par de nombreux incidents | Les Echos | 2013 |
| Rwanda : La campagne pour les législatives perturbée par de nombreux incidents | Les Echos | 2013 |
| Explosive Start to Rwandan Parliamentary Election | ThinkAfricaPress | 2013 |
| Rwanda: Un mort dans l'explosion d'une grenade à Kigali à 36 h des législatives (police) | Agence France Press | 2013 |
| Attentat à la grenade dans un marché de la capitale rwandaise... | Renseignor | 2013 |
| Rwanda - Rwanda: des opposants en exil responsables d'une attaque meurtrière à Kigali? | RFI | 2013 |
| Législatives rwandaises - Coup d'envoi explosif à Kigali | L'Observateur Paalga (Ouagadougou) | 2013 |
| Rwanda; 50 Injured in Kayonza District Road Accident | The New Times (Kigali) | 2013 |
| Rwanda; Two Dead, One Injured in Rubavu Accident | The New Times (Kigali) | 2014 |
| Rwanda; 18 Injured in Musanze Road Accident | The New Times (Kigali) | 2014 |
| Rwanda; Rubavu Accident Claims One, Injures Five | The New Times (Kigali) | 2014 |
| Rwanda; 14 Injured in Road Accident | The New Times (Kigali) | 2014 |
| Rwanda; Nation Mourns Gatsibo Road Accident Victims | The New Times (Kigali) | 2014 |
| Rwanda; Who Is to Blame for the Fatal Road Accidents? | The New Times (Kigali) | 2014 |
| Rwanda; 15 Passengers Killed, 24 Injured Following Eastern Province Bus Accident | The New Times (Kigali) | 2014 |
| Over 12 die in road accident in eastern Rwanda | BBC Monitoring Africa - Political | 2014 |
| Rwanda; Kiziguro Road Accident - All Injured Now Stable | Rwanda Focus (Kigali) | 2014 |
| Rwanda; Kiziguro Road Accident - All Injured Now Stable | Rwanda Focus (Kigali) | 2014 |
| Rwanda; Bride, Groom Involved in Road Accident Out of Danger - Medics | The New Times (Kigali) | 2014 |
| Rwanda; Bride, Groom Involved in Road Accident Out of Danger - Medics | The New Times (Kigali) | 2014 |
| Rwanda; Huye Road Accident Kills Four-Year-Old, Injures 13 | The New Times (Kigali) | 2014 |
| Rwanda; Weekend Accidents Claim 20 | The New Times (Kigali) | 2014 |
| Rwanda; Weekend Accidents Claim 20 | The New Times (Kigali) | 2014 |
| Rwanda; Murekezi Calls On Police to Do More About Road Accidents | Rwanda Focus (Kigali) | 2014 |
| Rwanda; Murekezi Calls On Police to Do More About Road Accidents | Rwanda Focus (Kigali) | 2014 |
| Une dizaine de morts et plusieurs blessés dans un accident de la route au sud du Rwanda | PANAPRESS - Pan African News Agency | 2014 |
| Rwanda; Murekezi Calls On Police to Do More About Road Accidents | Rwanda Focus (Kigali) | 2014 |
| Rwanda; Murekezi Calls On Police to Do More About Road Accidents | Rwanda Focus (Kigali) | 2014 |
| Rwanda RDF condemns soldiers shooting spree that left four people death | The New Times (Kigali) | 2014 |
| Les accidents de la route ont fait 37 morts en une semaine au Rwanda | PANAPRESS - Pan African News Agency | 2014 |
| Les accidents de la route ont fait 37 morts en une semaine au Rwanda | PANAPRESS - Pan African News Agency | 2014 |
| Rwanda; Two Killed in Christmas Morning Accident in City | The New Times (Kigali) | 2014 |
| Rwanda; Five Killed in Rulindo Bus Accident | The New Times (Kigali) | 2015 |
| Rwanda; Five Dead, Including Kigali Basketball Club Player, in Traffic Accident | Rwanda Focus (Kigali) | 2015 |
| Rwanda; Oil Tanker Crashes Into Home, Bursts into Flames | The New Times (Kigali) | 2015 |
| Rwanda; Student Killed, Five Injured in Kicukiro Accident | The New Times (Kigali) | 2015 |
| Rwanda; Seven Prisoners Die in Car Crash, Nine Others Injured | The New Times (Kigali) | 2015 |
| Rwanda; Seven Inmates Die in Karongi Accident | The New Times (Kigali) | 2015 |
| 5.8-magnitude quake injures 4 in Rwanda | Xinhua General News Service | 2015 |
| Six morts et des blessés dans un accident de la route à Rusizi | Rwanda News Agency | 2015 |
| Rwanda; One Dead, Nine Injured in Burera Road Accident | The New Times (Kigali) | 2015 |
| Grenade explosion in Rwanda kills1,injuries5 | Xinhua General News Service | 2015 |
| Rwanda; One Dead, 8 Injured in Rubavu Hospital Accident | The New Times (Kigali) | 2015 |
| 18 killed in road accident in Rwanda | Xinhua General News Service | 2015 |
| Eighteen killed as truck crashes into minibus in Rwanda | Deutsche Presse-Agentur | 2015 |
| Rwanda; Truck Crushes Minibus, Leaves 18 People Dead | The New Times (Kigali) | 2015 |
| Eighteen killed in road accident in Rwanda | BBC Monitoring Africa - Political | 2015 |
| Rwanda; Four Die, 15 Injured in Ngororero Road Accident | The New Times (Kigali) | 2015 |
| Quatre morts et quinze blessés dans un accident de la route à Ngororero | Rwanda News Agency | 2015 |
| Huit morts dans un accident de la route au nord du Rwanda | Rwanda News Agency | 2015 |
| Une attaque à la machette fait trois blessés dans un camp de réfugiés congolais au Rwanda | PANAPRESS - Pan African News Agency | 2015 |
| Rwanda; Two Killed, Ten Injured in Kigali-Gicumbi Road Accident | The New Times (Kigali) | 2016 |
| Rwanda; APR Handball Star Shimirwa Dies in an Accident | The New Times (Kigali) | 2016 |
| Quatre morts et plusieurs blessés dans un accident de la route à Huye | Rwanda News Agency | 2016 |
| Landslides kill nearly 50 in Rwanda | Digital Journal | 2016 |
| Landslides kill nearly 50 in Rwanda | Agence France Press | 2016 |
| Official: 49 people killed in Rwanda landslides amid rains; Official: 49 people killed in Rwanda landslides amid rains | Canadian Press | 2016 |
| 20 killed by Rwanda landslides | Africa Review | 2016 |
| 1ST LEAD | Deutsche Presse-Agentur | 2016 |
| Death toll from heavy rains, landslides rises to 49 in Rwanda | Xinhua General News Service | 2016 |
| Heavy rains trigger landslides in Rwanda, killing 49 | Deutsche Presse-Agentur | 2016 |
| 20 killed, mostly children, by Rwanda landslides | Agence France Press | 2016 |
| Death toll rises to 18 in Rwanda landslides | Xinhua General News Service | 2016 |
| Death toll from heavy rains in Rwanda climbs to 72 | Xinhua General News Service | 2016 |
| Dozens dead, hundreds homeless after landslides in northern Rwanda | dpa-AFX | 2016 |
| Official: 49 people killed in Rwanda landslides amid rains | The Associated Press | 2016 |
| Rwanda: Landslides kill 22 | Turkish Governmental News | 2016 |
| Heavy rains trigger landslides in Rwanda, killing 49 | dpa-AFX | 2016 |
| Nineteen die in Rwanda floods | BBC Monitoring Africa - Political | 2016 |
| Rwanda; Landslide Disaster Leaves 34 Dead | News of Rwanda | 2016 |
| Xinhua world news summary at 1530 GMT, May 9 | Xinhua General News Service | 2016 |
| Landslides In Northern Rwanda Kill 49 | International Business Times News | 2016 |
| Rwanda; Experts Weigh in On How Deadly Floods, Landslides Can Be Prevented | The New Times (Kigali) | 2016 |
| East Africa; El Nino-Induced Floods Ravage East Africa | Deutsche Welle | 2016 |
| Deadly Rwanda floods leave thousands homeless | Al Jazeera Africa | 2016 |
| 25 killed in Rwanda landslides | IANS-English | 2016 |
| Dozens killed in Rwanda landslides; World news in brief | The Independent | 2016 |
| 49 killed in landslides after Rwanda downpour; world in brief | The Evening Standard | 2016 |
| Rwanda; Floods Claim 49 Lives | Rwanda Focus | 2016 |
| 49 die in landslides in torrential rain | The Herald | 2016 |
| Rwanda; Survivors Recount Ordeal After Fatal City Road Accident | The New Times (Kigali) | 2016 |
| Rwanda; City Accident Victims Discharged | The New Times (Kigali) | 2016 |
| Rwanda; Seven Killed, Nine Injured in City Road Accident | The New Times (Kigali) | 2016 |
| Rwanda; Scores Killed in Kicukiro Nasty Accident | Rwanda Focus (Kigali) | 2016 |
| Un camion perd ses freins et provoque un accident mortel à Kigali | Rwanda News Agency | 2016 |
| Rwanda; Weekend Accident Claims One, Injures 27 in Rubavu | The New Times (Kigali) | 2016 |
| Rwanda; Four Die, Nine Injured in City Road Accident | The New Times (Kigali) | 2016 |
| Police kill three terror suspects in western Rwanda | Xinhua General News Service | 2016 |
| Rwanda; Volcano Passenger Bus Involved in Accident on Route to Kampala | Rwanda Focus (Kigali) | 2016 |
| Rwanda; 4.8 Magnitude Earthquake Kills One, Injures Over 20 | Rwanda Focus (Kigali) | 2016 |
| 4.8 magnitude quake hits western Rwanda - USGS | Xinhua General News Service | 2016 |
| Rwanda;four injuries and one dead in police shootout | The New Times (Kigali) | 2016 |
| Rwandan police officer kills colleague, injures three | Xinhua General News Service | 2016 |
| Xinhua world news summary at 0030 GMT, Oct. 3 | Xinhua General News Service | 2016 |
| Rwanda; Three RDF Soldiers Killed, 20 Injured in Truck Accident | The New Times (Kigali) | 2016 |
| Three RDF Soldiers Killed, 20 Injured in Truck Accident | The New Times (Kigali) | 2016 |
| Three RDF Soldiers Killed in Road Accident Laid to Rest | The New Times (Kigali) | 2016 |
| Rwanda; Three RDF Soldiers Killed in Road Accident Laid to Rest | The New Times (Kigali) | 2016 |
| Rwanda; Police Issue Warning Against Reckless Driving | The New Times (Kigali) | 2017 |
| Deadly Windly Wreak Havoc in Rwanda | CAJ News Agency | 2017 |
| Over 1,000 people displaced by floods in western Rwanda | BBC Monitoring Africa - Political | 2018 |
| 'Dead bodies everywhere' 16 killed and 140 injured as freak LIGHTNING strike hits church | Daily Star Onlien | 2018 |
| Heavy rains, landslides kill 18 in Rwanda | The Sun (Nigeria) | 2018 |
| Heavy Rains, Landslides Kill 18 in Rwanda | Africa Newswire | 2018 |
| Fatal Landslides | Daily News (South Africa) | 2018 |
| Rwanda's recurring landslides worse this year with over 200 people dead | Ventures Africa | 2018 |
| At least 12 dead, several missing after landslide in Rwanda | dpa international (Englischer Dienst) | 2018 |
| Landslides kill 18 in Rwanda, death toll at 200 since January | The Daily Monitor | 2018 |
| Latest Rwandan landslides kill at least 18 people | UPI | 2018 |
| Mudslide kills 12 | Daily News (South Africa) | 2018 |
| Landslides leave 18 dead | The Herald | 2018 |
| Landslides leave 18 dead | The Herald (Glasgow) | 2018 |
| Rains wreak havoc in Rwanda, 18 killed | Anadolu Agency (AA) | 2018 |
| Flood deaths; GLOBAL BRIEFING | Daily Post (North Wales) | 2018 |
| Official: 18 killed amid flooding, landslides in Rwanda | The Associated Press | 2018 |
| Landslides in Western Rwanda kill 18 People After Heavy Rains on Weekend | Sputnik News Service | 2018 |
| Rain tolls upp by 18 | The Sun | 2018 |
| No Headline In Original | Midi Libre | 2018 |
| Rwanda Mourns Deaths of 14 Miners as Official Urges More Safety Steps | Voice of America News | 2019 |
| 14 Killed in Landslide | The Sun | 2019 |
| 18 dead in heavy floods in Rwanda | Global English | 2019 |
| Heavy Rainfall in Rwanda leaves 10 dead | Global English | 2019 |
| 8 dead, 17 injured in road accident in western Rwanda | Xinhua General News Service | 2019 |
| Eight killed, 18 hurt in Rwanda attack | The Mercury (South Africa) | 2019 |
| Unknown assailants kill eight people near Rwanda's volcanoes park | Iran Daily | 2019 |
| Eight killed,18 wounded in Rwanda attack | The Daily Monitor | 2019 |
| Eight killed,18 wounded in Rwanda attack | Daily Nation (Kenya) | 2019 |
| Unknown assailants kill eight people near Rwanda's volcanoes park | Iran Daily | 2019 |
| 8 killed at tourist spot | Daily Record and Sunday Mail | 2019 |
| Attaque mortelle au Rwanda | Ouest-France | 2019 |
| 13 killed in Rwanda landslides | Xinhua General News Service | 2020 |
| 13 killed in Rwanda landslides | Xinhua General News Service | 2020 |
| Death toll climbs to 19 in Rwanda's heavy rains | Xinhua General News Service | 2020 |
| Landslides kill 13, wound 2 in Rwanda | Global English (Middle East and North Africa Financial Network) | 2020 |
| 7 killed, 8 injured in road accident in Rwanda | Xinhua General News Service | 2020 |
| At least 65 killed in flooding, landslides in Rwanda; At least 65 killed in flooding, landslides in Rwanda | Canadian Press | 2020 |
| Texas salon owner jailed for defying governor's order freed NEWS BRIEFING Staff and news services Virus hospitalization is new barrier to military enlistment White father and son arrested in shooting death of black man GOP fundraiser and political donor to be postmaster general Rwanda flooding, landslides leave at least 65 dead Google scraps smart-city development for Toronto | The Hartford Courant | 2020 |
| Texas salon owner jailed for defying governor's order freed Virus hospitalization is new barrier to military enlistment Advocates: More immigrant deaths without Trump action GOP fundraiser and political donor to be postmaster general Rwanda flooding, landslides leave at least 65 dead Google scraps smart-city development for Toronto | The Morning Call | 2020 |
| Floods kill 65 in Rwanda as heavy rains pound East Africa | Agence France Press | 2020 |
| Flooding and landslides kill at least 65 in northern Rwanda; An estimated 11,000 people have been affected by heavy rain this year and 1,000 hectares of crops have been destroyed | The Telegraph | 2020 |
| 1st LD Writethru: 55 people killed as heavy rains batter Rwanda | Xinhua General News Service | 2020 |
| Urgent: 55 people killed in Rwanda heavy rains | Xinhua General News Service | 2020 |
| Rwanda: Torrential rains kill scores in Rwanda | Thai News Service | 2020 |
| 55 die in Rwandan floods, mudslides | Cape Argus (South Africa) | 2020 |
| In brief | Northern Advocate (New Zealand) | 2020 |
| Flash Flooding Kills 55 Across Rwanda - Reports | Sputnik News Service | 2020 |
| More than 70 dead in Rwanda after floods, landslides | UPI | 2020 |
| At least 65 killed in Rwanda flooding, landslides | The Indian Awaaz | 2020 |
