# Supplementary material for: Systematic media review: A novel method to assess mass-trauma epidemiology in absence of databases—A pilot-study in Rwanda
Source: PLoS One. 2021 Oct 13;16(10):e0258446. doi: 10.1371/journal.pone.0258446 (PMC8513851; doi:10.1371/journal.pone.0258446)
Supplement: S4 Appendix — (DOCX) [file pone.0258446.s004.docx]

**Appendix 4.** All mass-trauma events identified in the media review.

| **Type of injury** | **Province** | **District(s)** | **Number of on-site deaths** | **Number of people injured** | **Hospital(s) specified in article** | **Date (YY-MM-DD)** | **Hospital level** |
| --- | --- | --- | --- | --- | --- | --- | --- |
| RTA | Southern | Huye | 3 | 35 | Yes | 2010-02-12 | Tertiary hospital |
| RTA | Southern | - | 1 | 24 | Yes | 2010-02-14 | Tertiary hospital, district hospital |
| Violence/ terrorism | Kigali | - | 0 | 16 | No | 2010-03-05 | - |
| Natural hazard | Western | Nyabihu, Rubavu | 4 | 17 | No | 2010-03-26 | - |
| Natural hazard | Western | Rutsiro | 20 | 19 | Yes | 2010-04-07 | Provincial hospital |
| RTA | Kigali | Nyarugenge | 0 | 13 | Yes | 2010-04-08 | Tertiary hospital |
| RTA | Eastern | Rwamagana | 0 | 35 | Yes | 2010-05-02 | Tertiary hospital, provincial hospital |
| RTA | Western | Karongi | 0 | 4 | Yes | 2010-05-02 | Health center |
| RTA | Northern | Gakenke | 0 | 12 | No | 2010-05-06 | - |
| RTA | Northern | - | 0 | 12 | Yes | 2010-05-06 | District hospital |
| Violence/ terrorism | Kigali | Kicukiro, Nyarugenge | 2 | 32 | Yes | 2010-05-15 | - |
| RTA | Kigali | Kicukiro | 7 | 9 | Yes | 2010-06-10 | Tertiary hospital |
| Violence/ terrorism | Kigali | Kicukiro | 0 | 27 | Yes | 2010-08-11 | Tertiary hospital |
| Violence/ terrorism | Kigali | - | - | 7 | No | 2010-08-13 | - |
| RTA | Eastern | Rwamagana | 25 | 0 | Yes | 2010-10-09 | Provincial hospital |
| RTA | Southern | Gisagara | 3 | 13 | Yes | 2010-11-27 | Tertiary hospital |
| Natural hazard | Eastern | Ngoma | 6 | - | No | 2010-12-03 | - |
| RTA | Southern | Kamonyi | 0 | 6 | Yes | 2010-12-30 | Tertiary hospital |
| Violence/ terrorism | Kigali | Nyarugenge | 0 | 7 | No | 2011-01-03 | Tertiary hospital |
| Violence/ terrorism | Kigali | Gasabo | 2 | 28 | No | 2011-01-28 | - |
| RTA | Northern | Rulindo | 1 | 19 | Yes | 2011-02-17 | Tertiary hospital |
| Violence/ terrorism | Kigali | Nyarugenge | 0 | 10 | Yes | 2011-03-01 | Tertiary hospital |
| Boat accident | Western | Rubavu | 5 | 37 | No | 2011-05-19 | - |
| RTA | Eastern | Nyagatare | 0 | 36 | Yes | 2011-06-05 | Tertiary hospital, District hospital |
| RTA | Western | Ngororero | 10 | 7 | Partly | 2011-07-01 | Tertiary hospital, District hospital |
| RTA | Northern | Rulindo | 0 | 5 | No | 2011-07-11 | - |
| Violence/ terrorism | Western | Rusizi | 0 | 21 | Yes | 2011-07-12 | District hospital |
| RTA | Kigali | Gasabo | 0 | 15 | Yes | 2011-08-02 | Tertiary hospital, district hospital |
| Natural hazard | Western | Nyabihu | 3 | - | Yes | 2011-08-02 | Health center |
| RTA | Southern | Huye | 0 | 5 | Yes | 2011-09-09 | District hospital |
| Natural hazard | Western | Nyabihu | 1 | 16 | Yes | 2011-09-29 | District hospital, Health center |
| Natural hazard | Western | Karongi | 10 | - | No | 2011-11-04 | - |
| Natural hazard | Southern | Muhanga | 3 | - | No | 2011-11-10 | - |
| RTA | Northern | Rulindo | 2 | 3 | Yes | 2011-12-25 | Tertiary hospital |
| Violence/ terrorism | Kigali | Gasabo | 2 | 18 | Yes | 2012-01-03 | Tertiary hospital, District hospital |
| Violence/ terrorism | Southern | Muhanga | 0 | 10 | No | 2012-01-24 | - |
| RTA | Southern | Huye | 0 | 3 | Yes | 2012-01-25 | Tertiary hospital |
| RTA | Northern | Musanze | 0 | 6 | Yes | 2012-02-25 | Provincial hospital |
| RTA | Southern | Kamonyi | 8 | 30 | Yes | 2012-03-16 | Tertiary hospital, District hospital |
| Violence/ terrorism | Northern | Musanze | 1 | 5 | No | 2012-03-23 | - |
| Violence/ terrorism | Kigali | Nyarugenge | 0 | 6 | No | 2012-03-30 | - |
| Natural hazard | Northern, Western | Nyabihu, Musanze, Rubavu | 5 | - | No | 2012-04-11 | - |
| Natural hazard | Northern, Western | Musanze, Rubavu | 8 | - | No | 2012-04-16 | - |
| RTA | Western | Rubavu | 2 | 11 | Yes | 2012-05-10 | District hospital |
| RTA | Southern | Ruhango | 8 | 15 | Yes | 2012-06-27 | District hospital, Health center |
| RTA | Western | Rubavu | 5 | 8 | No | 2012-07-12 | Tertiary hospital, District hospital |
| Natural hazard | Western | Rubavu | 1 | - | No | 2012-09-08 | - |
| RTA | Kigali | Kicukiro | 1 | 6 | Yes | 2012-09-09 | Tertiary hospital |
| RTA | Southern | Kamonyi | 2 | 10 | Yes | 2012-12-17 | Tertiary hospital |
| RTA | Southern | Nyanza | 4 | 5 | Yes | 2013-01-04 | Tertiary hospital |
| RTA | Northern | Musanze | 1 | 6 | Yes | 2013-01-28 | Provincial hospital |
| RTA | Southern | Kamonyi | 0 | 15 | Yes | 2013-03-25 | Tertiary hospital |
| Violence/ terrorism | Kigali | Gasabo | 1 | 8 | Yes | 2013-03-26 | District hospital |
| RTA | Southern | Huye | 6 | 20 | Yes | 2013-05-03 | Tertiary hospital |
| RTA | Southern | Nyanza | 0 | 15 | No | 2013-06-05 | - |
| RTA | Eastern | Nyagatare | 5 | - | No | 2013-06-25 | - |
| RTA | Eastern | Kirehe | 6 | 16 | Yes | 2013-07-18 | Tertiary hospital, District hospital |
| RTA | Western | Rusizi | 5 | 12 | No | 2013-07-18 | - |
| Violence/ terrorism | Kigali | Nyarugenge | 3 | 32 | No | 2013-07-26 | - |
| RTA | Western | Rusizi | 2 | 8 | Yes | 2013-08-04 | District hospital |
| Natural hazard | Eastern | Nyagatare | 2 | 4 | No | 2013-09-01 | - |
| Violence/ terrorism | Kigali | Kicukiro | 1 | 14 | No | 2013-09-14 | - |
| RTA | Eastern | Kayonza | - | 50 | Partly | 2013-10-29 | Tertiary hospital, District hospital |
| RTA | Western | Rubavu | 2 | 1 | Yes | 2014-01-30 | District hospital |
| RTA | Northern | Musanze | 0 | 18 | Yes | 2014-02-12 | Provincial hospital, District hospital |
| RTA | Western | Rubavu | 1 | 5 | Yes | 2014-04-02 | Tertiary hospital, District hospital |
| RTA | Kigali | Gasabo, Kicukiro | 0 | 14 | No | 2014-04-23 | Tertiary hospital, Provincial hospital |
| RTA | Eastern | Gatsibo | 15 | 24 | Yes | 2014-07-22 | Tertiary hospital, District hospital |
| RTA | Southern | Huye | 0 | 4 | Yes | 2014-07-27 | Tertiary Hospital, District hospital |
| RTA | Southern | Huye | 1 | 13 | Yes | 2014-08-01 | Tertiary |
| RTA | Southern | Kamonyi | 12 | 14 | Yes | 2014-08-09 | Tertiary Hospital |
| RTA | Northern | Gakenke | 7 | - | No | 2014-08-09 | - |
| RTA | Southern | Huye | 1 | 3 | No | 2014-08-09 | - |
| RTA | Southern | Muhanga, Kamonyi | 12 | 13 | No | 2014-08-09 | - |
| RTA | Northern | Musanze | 3 | - | No | 2014-08-10 | - |
| RTA | Kigali | Nyarugenge | 7 | 10 | No | 2014-08-10 | - |
| Violence/ terrorism | Northern | Gicumbi | 4 | 7 | Yes | 2014-08-10 | Tertiary Hospital |
| RTA | Northern | Gakenke | 5 | 21 | No | 2014-08-10 | - |
| RTA | Southern | Muhanga | 12 | - | No | 2014-08-10 | - |
| RTA | Kigali | Kicukiro | 2 | 4 | No | 2014-12-25 | - |
| RTA | Northern | Rulindo | 3 | 18 | No | 2015-02-07 | - |
| RTA | Southern | Kamonyi | 5 | 15 | Yes | 2015-03-01 | Tertiary hospital, District hospital |
| RTA | Western | Nyabihu | 3 | 1 | Yes | 2015-06-25 | Health center |
| RTA | Kigali | Kicukiro | 1 | 5 | Yes | 2015-07-09 | Tertiary hospital, District hospital |
| RTA | Western | Karongi | 7 | 12 | No | 2015-07-09 | Tertiary hospital, Provincial hospital |
| Natural hazard | Not mentioned | Not mentioned | 0 | 4 | No | 2015-08-07 | - |
| RTA | Western | Rusizi | 6 | - | Yes | 2015-08-19 | District hospital |
| RTA | Northern | Burera | 1 | 9 | Yes | 2015-08-24 | Provincial hospital |
| Violence/ terrorism | Kigali | Gasabo | 1 | 5 | Yes | 2015-08-29 | District hospital |
| RTA | Western | Rubavu | 1 | 8 | Yes | 2015-09-03 | Tertiary hospital, District hospital |
| RTA | Eastern | Rwamagana | 18 | 2 | Yes | 2015-09-21 | Tertiary hospital |
| RTA | Western | Ngororero | 4 | 15 | Yes | 2015-10-01 | Tertiary hospital |
| RTA | Northern | Gicumbi | 8 | 2 | Yes | 2015-10-31 | Tertiary hospital |
| Violence/ terrorism | Western | Karongi | 0 | 3 | No | 2015-12-24 | - |
| RTA | Northern | Gicumbi | 1 | 11 | Yes | 2016-01-18 | District hospital |
| RTA | Southern | Huye | 4 | 4 | Yes | 2016-04-22 | Tertiary hospital |
| Natural hazard | Southern, Northern, Western | Gakenke, Muhanga, Rubavu, Ngororero | 72 | 26 | No | 2016-05-07 | - |
| RTA | Kigali | Kicukiro | 7 | 9 | Yes | 2016-06-10 | Tertiary hospital |
| RTA | Western | Rubavu | 1 | 27 | Yes | 2016-06-13 | District hospital |
| RTA | Kigali | Nyarugenge | 4 | 9 | Yes | 2016-06-31 | Tertiary hospital |
| RTA | Eastern | Nyagatare | - | - | Yes | 2016-09-19 | District hospital |
| Natural hazard | Western | Rusizi | 1 | 20 | Yes | 2016-09-23 | District hospital |
| Violence/ terrorism | Eastern | Gatsibo | 1 | 3 | Yes | 2016-10-02 | District hospital |
| RTA | Kigali | Kicukiro | 3 | 21 | Yes | 2016-10-11 | Tertiary hospital |
| RTA | Kigali | Gasabo | 4 | - | No | 2017-04-18 | - |
| Natural hazard | Northern, Southern, Eastern, Western | Gicumbi, Huye, Bugesera, Kirehe, Ngoma, Nyabihu, Nyamasheke, Rubavu, Rusizi | 3 | 24 | No | 2017-10-05 | - |
| Natural hazard | Western | Rubavu | 0 | 3 | No | 2018-03-03 | - |
| Natural hazard | Southern | Nyaruguru | 16 | 140 | No | 2018-03-10 | - |
| Natural hazard | Northern, Kigali, Eastern | Gatsibo, other (not specified) | 18 | - | No | 2018-04-23 | - |
| Natural hazard | Northern, Western | Karongi, Rubavu | 18 | 12 | No | 2018-05-06 | - |
| Natural hazard | Eastern | Rwamagana | 14 | - | No | 2019-01-22 | - |
| Natural hazard | Western | Karongi | 15 | - | No | 2019-05-19 | - |
| RTA | Western | Karongi | 8 | 17 | Yes | 2019-06-16 | Tertiary hospital, Provincial hospital |
| Violence/ terrorism | Northern | Musanze | 8 | 18 | No | 2019-10-05 | - |
| Natural hazard | Kigali, Eastern | Gatsibo, Gasabo, Kicukiro, Nyarugenge | 19 | 8 | No | 2020-02-02 | - |
| RTA | Southern | Kamonyi | 7 | 8 | No | 2020-02-13 | - |
| Natural hazard | Southern, Northern, Western | Gakenke, Nyabihu, Muhanga | 65 | - | No | 2020-05-07 | - |

RTA = Road traffic accidents.
