# Supplementary material for: Systematic media review: A novel method to assess mass-trauma epidemiology in absence of databases—A pilot-study in Rwanda
Source: PLoS One. 2021 Oct 13;16(10):e0258446. doi: 10.1371/journal.pone.0258446 (PMC8513851; doi:10.1371/journal.pone.0258446)
Supplement: S5 Appendix — (DOCX) [file pone.0258446.s005.docx]

**Appendix 5.** Proposed data collection form for systematic media reviews.

- **Article title:** __________
- **Who published article?** (newspaper name) __________
- **Language of article:** English / Other (adapt to relevant context)
- **Date of publication (YYMMDD):** __________
- **Date of trauma event (YYMMDD):** __________
- **Geographical location of the event:** adapt to relevant context
- **What was the mechanism of trauma?** Road traffic accident / natural hazards / Act of violence/terrorism / other
  - If “road traffic accident”:
    - **What type of vehicles/persons were involved in the road traffic accident (check all that apply)?** Bus, car or truck / motorcycle / bicycle / pedestrians / other
    - **What happened in the accident?** Collision / derailment (slid off the road) / Other
  - If “natural hazard”:
    - **What type of natural hazard was it?** Landslide or flooding / earthquake / storm, hurricane or cyclone / other
  - If “Act of Violence/ terrorism”:
    - **What type of act of Violence/ terrorism-related mechanism was it?** Machete or knife / gun, rifle, or firearm / explosion or detonation / other
- **Number of persons injured (not dead; including mild and critical injuries):** __________
- **Number of persons critically injured (if specified):** ___________
- **Number of on-site deaths:** __________
- **To what extent was demographic data (names, age, gender, nationality) provided**? None / data for some of the deceased only / data for all the deceased only / data for some of the victims (injured and deceased) / data for all victims (injured and deceased)
- **Demographic data provided (check all that apply):** names of victims / gender / age / age-group (e.g. “children”, “students” or “elderly”) / none / other
- **Was there information on to which health facilities the victims were taken?** Yes / no / partially
- **To what level of health facility were the victim(s) taken (check all that apply)?** Health center / district hospital / provincial hospital / tertiary hospital
- **Name of hospital patients were taken to:** __________
- **Any other relevant information shared:** __________
