# Supplementary material for: Systematic media review: A novel method to assess mass-trauma epidemiology in absence of databases—A pilot-study in Rwanda
Source: PLoS One. 2021 Oct 13;16(10):e0258446. doi: 10.1371/journal.pone.0258446 (PMC8513851; doi:10.1371/journal.pone.0258446)
Supplement: S1 Raw data — (PDF) [file pone.0258446.s006.pdf]

Epidemiology of mass trauma in Rwanda PID 1755

Codebook

Data Dictionary Codebook

2021-08-30 16:54:06

Collapse all instruments

#	Variable / Field Name	Field Label *Field Note*	Field Attributes (Field Type, Validation, Choices, Calculations, etc.)
Instrument: **Systematic media review** (systematic_media_review) Enabled as survey			Collapse
1	data_collector_initials	Initials of data collector	text, Required
2	article_title	Article title	text, Required
3	article_publisher	Who published the article? (newspaper name)	text, Required
4	article_langugage	Language of article	radio, Required<br>1 English<br>2 French<br>3 Kinyarwanda<br>4 Other
5	article_date	Date of publication (year-month-date, e.g. 2012-02-20)	text (date_ymd), Required
6	event_date	Date of trauma event (year-month-date, e.g. 2012-02-20)	text (date_ymd), Required
7	event_location_province	Geographical location of event - which province did it occur in?	radio, Required<br>1 Northern<br>2 Western<br>3 Southern<br>4 Eastern<br>5 Kigali<br>6 Not mentioned / other

8	event_location_sector	In which sector did it occur? (pick all applicable)	checkbox, Required<br>1 event_location_sector___1 Gasabo (Kigali)<br>2 event_location_sector___2 Kicukiro (Kigali)<br>3 event_location_sector___3 Nyarugenge (Kigali)<br>4 event_location_sector___4 Burera (Northern)<br>5 event_location_sector___5 Gakenke (Northern)<br>6 event_location_sector___6 Gicumbi (Northern)<br>7 event_location_sector___7 Musanze (Northern)<br>8 event_location_sector___8 Rulindo (Northern)<br>9 event_location_sector___9 Gisagara (Southern)<br>10 event_location_sector___10 Huye (Southern)<br>11 event_location_sector___11 Kamonyi (Southern)<br>12 event_location_sector___12 Muhanga (Southern)<br>13 event_location_sector___13 Nyamagabe (Southern)<br>14 event_location_sector___14 Nyanza (Southern)<br>15 event_location_sector___15 Nyaruguru (Southern)<br>16 event_location_sector___16 Ruhango (Southern)<br>17 event_location_sector___17 Bugesera (Eastern)<br>18 event_location_sector___18 Gatsibo (Eastern)<br>19 event_location_sector___19 Kayonza (Eastern)<br>20 event_location_sector___20 Kirehe (Eastern)<br>21 event_location_sector___21 Ngoma (Eastern)<br>22 event_location_sector___22 Nyagatare (Eastern)<br>23 event_location_sector___23 Rwamagana (Eastern)<br>24 event_location_sector___24 Karongi (Western)<br>25 event_location_sector___25 Ngororero (Western)<br>26 event_location_sector___26 Nyabihu (Western)<br>27 event_location_sector___27 Nyabihu (Western)<br>28 event_location_sector___28 Nyamasheke (Western)<br>29 event_location_sector___29 Rubavu (Western)<br>30 event_location_sector___30 Rusizi (Western)<br>31 event_location_sector___31 Rutsiro (Western)<br>32 event_location_sector___32 Other / Unknown
9	event_mechanism	What was the mechanism of trauma?	radio, Required<br>1 Road traffic accident<br>2 Natural hazard event<br>3 Man-made trauma<br>4 Other

10	event_mechanism_rta_vehicles<br>Show the field ONLY if:<br>[event_mechanism] = '1'	What type of vehicles/persons were involved in the road traffic accident? (check all that apply)	checkbox, Required<br>1 event_mechanism_rta_vehicles___1 Bus<br>2 event_mechanism_rta_vehicles___2 Moto<br>3 event_mechanism_rta_vehicles___3 Car<br>4 event_mechanism_rta_vehicles___4 Truck<br>5 event_mechanism_rta_vehicles___5 Bicycle<br>6 event_mechanism_rta_vehicles___6 Pedestrians<br>7 event_mechanism_rta_vehicles___7 Other
11	event_mechanism_rta_vehicles_other<br>Show the field ONLY if:<br>[event_mechanism_rta_vehicles(7)] = '1'	If other, please explain	text, Required
12	event_mechanism_rta_mechanism<br>Show the field ONLY if:<br>[event_mechanism] = '1'	What happened in the road traffic accident?	radio, Required<br>1 Collision<br>2 Derailment (slid off the road)<br>3 Other
13	event_mechanism_rta_mechanism_other<br>Show the field ONLY if:<br>[event_mechanism_rta_mechanism] = '3'	If other, please explain	text, Required
14	event_mechanism_natural<br>Show the field ONLY if:<br>[event_mechanism] = '2'	What type of natural hazard event was it?	radio, Required<br>1 Landslide / flooding<br>2 Earthquake<br>3 Storm / hurricane / cyclone<br>4 Other
15	event_mechanism_natural_other<br>Show the field ONLY if:<br>[event_mechanism_natural] = '4'	If other, please explain	text, Required
16	event_mechanism_manmade<br>Show the field ONLY if:<br>[event_mechanism] = '3'	What type of man-made injury was it?	radio, Required<br>1 Machete / knife<br>2 Gun / rifle / firearm<br>3 Explosion / detonation<br>4 Other
17	event_mechanism_manmade_other<br>Show the field ONLY if:<br>[event_mechanism_manmade] = '4'	If other, please explain	text, Required
18	event_victims	Number of trauma victims (injured or potentially injured, NOT dead)	text (integer), Required
19	event_casualties	Number of on-site casualties (NOT victims who died in the hospital)	text (integer), Required
20	event_demography	Share any demographic details provided (e.g. number of men/women, number of children/adults, the age of the victims)	text, Required
21	systematic_media_review_complete	Section Header: *Form Status*<br>Complete?	dropdown<br>0 Incomplete<br>1 Unverified<br>2 Complete
