# Supplementary material for: Systematic media review: A novel method to assess mass-trauma epidemiology in absence of databases—A pilot-study in Rwanda
Source: PLoS One. 2021 Oct 13;16(10):e0258446. doi: 10.1371/journal.pone.0258446 (PMC8513851; doi:10.1371/journal.pone.0258446)
Supplement: Attachment — Submitted filename: PONE-D-21-19748-R.pdf [file pone.0258446.s008.pdf]

PLOS ONE

Systematic media review: a novel method to assess mass-trauma epidemiology in absence of databases - a pilot-study in Rwanda.

--Manuscript Draft--

**Manuscript Number:**	PONE-D-21-19748
**Article Type:**	Research Article
**Full Title:**	Systematic media review: a novel method to assess mass-trauma epidemiology in absence of databases - a pilot-study in Rwanda.
**Short Title:**	Systematic media review: a pilot-study of mass-trauma epidemiology in Rwanda
**Corresponding Author:**	Lotta Velin<br>Linköping University: Linkopings universitet<br>Båstad, SWEDEN
**Keywords:**	
**Abstract:**	Objective Surge capacity refers to preparedness of health systems to face sudden patient inflows, such as mass-casualty incidents (MCI). To strengthen surge capacity, it is essential to understand MCI epidemiology, which is poorly studied in low- and middle-income countries lacking trauma databases. We propose a novel approach, the "systematic media review", to analyze mass-trauma epidemiology; here piloted in Rwanda. Methods A systematic media review of non-academic publications of MCIs in Rwanda between January 1 st , 2010, and September 1 st , 2020 was conducted using NexisUni. All articles identified by the search strategy were screened using eligibility criteria. Data were extracted in a RedCap form and analyzed using descriptive statistics. Findings Of 3187 articles identified, 247 met inclusion criteria. In total, 117 MCIs were described, of which 73 (62.4%) were road-traffic accidents, 23 (19.7%) natural hazards, 20 (17.1%) acts of violence/terrorism, and 1 (0.09%) boat collision. Of Rwanda's 30 Districts, 29 were affected by mass-trauma, with the rural Western province most frequently affected. Road-traffic accidents was the leading MCI until 2017 when natural hazards became most common. The median number of injured persons per event was 11 (IQR 5-18), and median on-site deaths was 2 (IQR 1-6); with natural hazards having the highest median deaths (6 [IQR 2-18]). Conclusion In Rwanda, MCIs have decreased, although landslides/floods are increasing, preventing a decrease in trauma-related mortality. By training journalists in "mass-casualty reporting", the potential of the "systematic media review" could be further enhanced, as a way to collect MCI data in settings without databases.
**Order of Authors:**	Lotta Velin
	Mbonyintwari Donatien
	Andreas Wladis
	Menelas Nkeshimana
	Robert Riviello
	Jean-Marie Uwitonze
	Jean-Claude Byiringiro
	Faustin Ntirenganya
	Laura Pompermaier
**Additional Information:**	
**Question**	**Response**
**Financial Disclosure**<br><br>Enter a financial disclosure statement that describes the sources of funding for the	The author(s) received no specific funding for this work.

Powered by Editorial Manager® and ProduXion Manager® from Aries Systems Corporation

work included in this submission. Review the submission guidelines for detailed requirements. View published research articles from *PLOS ONE* for specific examples.<br><br>This statement is required for submission and **will appear in the published article** if the submission is accepted. Please make sure it is accurate.<br><br>**Unfunded studies**<br>Enter: *The author(s) received no specific funding for this work.*<br><br>**Funded studies**<br>Enter a statement with the following details:<br>• Initials of the authors who received each award<br>• Grant numbers awarded to each author<br>• The full name of each funder<br>• URL of each funder website<br>• Did the sponsors or funders play any role in the study design, data collection and analysis, decision to publish, or preparation of the manuscript?<br>• **NO** - Include this sentence at the end of your statement: *The funders had no role in study design, data collection and analysis, decision to publish, or preparation of the manuscript.*<br>• **YES** - Specify the role(s) played.<br><br>* typeset	
**Competing Interests**<br><br>Use the instructions below to enter a competing interest statement for this submission. On behalf of all authors, disclose any competing interests that could be perceived to bias this work—acknowledging all financial support and any other relevant financial or non-financial competing interests.<br><br>This statement is required for submission and **will appear in the published article** if the submission is accepted. Please make sure it is accurate and that any funding sources listed in your Funding Information	The authors have declared that no competing interests exist.

later in the submission form are also declared in your Financial Disclosure statement.<br><br>View published research articles from *PLOS ONE* for specific examples.<br><br>**NO authors have competing interests**<br><br>Enter: *The authors have declared that no competing interests exist.*<br><br>**Authors with competing interests**<br><br>Enter competing interest details beginning with this statement:<br><br>*I have read the journal's policy and the authors of this manuscript have the following competing interests: [insert competing interests here]*<br><br>* typeset	
**Ethics Statement**<br><br>Enter an ethics statement for this submission. This statement is required if the study involved:<br><br>• Human participants<br>• Human specimens or tissue<br>• Vertebrate animals or cephalopods<br>• Vertebrate embryos or tissues<br>• Field research<br><br>Write "N/A" if the submission does not require an ethics statement.<br><br>General guidance is provided below. Consult the submission guidelines for detailed instructions. **Make sure that all information entered here is included in the Methods section of the manuscript.**	N/A

**Format for specific study types**<br><br>**Human Subject Research (involving human participants and/or tissue)**<br>• Give the name of the institutional review board or ethics committee that approved the study<br>• Include the approval number and/or a statement indicating approval of this research<br>• Indicate the form of consent obtained (written/oral) or the reason that consent was not obtained (e.g. the data were analyzed anonymously)<br><br>**Animal Research (involving vertebrate animals, embryos or tissues)**<br>• Provide the name of the Institutional Animal Care and Use Committee (IACUC) or other relevant ethics board that reviewed the study protocol, and indicate whether they approved this research or granted a formal waiver of ethical approval<br>• Include an approval number if one was obtained<br>• If the study involved *non-human primates*, add *additional details* about animal welfare and steps taken to ameliorate suffering<br>• If anesthesia, euthanasia, or any kind of animal sacrifice is part of the study, include briefly which substances and/or methods were applied<br><br>**Field Research**<br><br>Include the following details if this study involves the collection of plant, animal, or other materials from a natural setting:<br>• Field permit number<br>• Name of the institution or relevant body that granted permission	
**Data Availability**<br><br>Authors are required to make all data underlying the findings described fully available, without restriction, and from the time of publication. PLOS allows rare exceptions to address legal and ethical concerns. See the PLOS Data Policy and FAQ for detailed information.	Yes - all data are fully available without restriction

A Data Availability Statement describing where the data can be found is required at submission. Your answers to this question constitute the Data Availability Statement and **will be published in the article**, if accepted.<br><br>**Important:** Stating 'data available on request from the author' is not sufficient. If your data are only available upon request, select 'No' for the first question and explain your exceptional situation in the text box.<br><br>Do the authors confirm that all data underlying the findings described in their manuscript are fully available without restriction?	
**Describe where the data may be found in full sentences. If you are copying our sample text, replace any instances of XXX with the appropriate details.**<br><br>• If the data are **held or will be held in a public repository**, include URLs, accession numbers or DOIs. If this information will only be available after acceptance, indicate this by ticking the box below. For example: *All XXX files are available from the XXX database (accession number(s) XXX, XXX.).*<br>• If the data are all contained **within the manuscript and/or Supporting Information files**, enter the following: *All relevant data are within the manuscript and its Supporting Information files.*<br>• If neither of these applies but you are able to provide **details of access elsewhere**, with or without limitations, please do so. For example:<br><br>*Data cannot be shared publicly because of [XXX]. Data are available from the XXX Institutional Data Access / Ethics Committee (contact via XXX) for researchers who meet the criteria for access to confidential data.*<br><br>*The data underlying the results presented in the study are available from (include the name of the third party*	The data underlying the results presented in the study are available from Nexis Uni.

and contact information or URL).<br>• This text is appropriate if the data are owned by a third party and authors do not have permission to share the data.<br><br>* typeset	
Additional data availability information:	

Manuscript

Click here to access/download;Manuscript;Media review_final.docx

Systematic media review: a novel method to assess mass-trauma epidemiology in absence of databases - a pilot-study in Rwanda.

Lotta Velin[1], Mbonyintwari Donatien[2], Andreas Wladis[1], Menelas Nkeshimana[3], Robert Riviello[4,5], Jean-Marie Uwitonze[6], Jean-Claude Byiringiro[2], Faustin Ntirenganya[2,3], Laura Pompermaier[1,5]

1. Center for Teaching & Research in Disaster Medicine and Traumatology (KMC), Department of Biomedical and Clinical Sciences, Linköping University, Linköping, Sweden
2. College of Medicine and Health Sciences, University of Rwanda, Kigali, Rwanda
3. University Teaching Hospital in Kigali, Kigali, Rwanda
4. Brigham and Women's Hospital, Boston, MA, USA
5. Program in Global Surgery and Social Change, Department of Global Health an Social Medicine, Harvard Medical School, Boston, MA, USA
6. Emergency Medical Services (SAMU), Kigali, Rwanda

Corresponding author:

Lotta Velin, lotta.velin@liu.se

Katastrofmedicinskt Centrum, Johannes Magnus väg 11, 583 30 Linköping, Sweden

Word count: 2999/3000

Abstract word count: 249/250

Abstract

Objective

Surge capacity refers to preparedness of health systems to face sudden patient inflows, such as mass-casualty incidents (MCI). To strengthen surge capacity, it is essential to understand MCI epidemiology, which is poorly studied in low- and middle-income countries lacking trauma databases. We propose a novel approach, the “systematic media review”, to analyze mass-trauma epidemiology; here piloted in Rwanda.

Methods

A systematic media review of non-academic publications of MCIs in Rwanda between January 1st, 2010, and September 1st, 2020 was conducted using NexisUni. All articles identified by the search strategy were screened using eligibility criteria. Data were extracted in a RedCap form and analyzed using descriptive statistics.

Findings

Of 3187 articles identified, 247 met inclusion criteria. In total, 117 MCIs were described, of which 73 (62.4%) were road-traffic accidents, 23 (19.7%) natural hazards, 20 (17.1%) acts of violence/terrorism, and 1 (0.09%) boat collision. Of Rwanda’s 30 Districts, 29 were affected by mass-trauma, with the rural Western province most frequently affected. Road-traffic accidents was the leading MCI until 2017 when natural hazards became most common. The median number of injured persons per event was 11 (IQR 5-18), and median on-site deaths was 2 (IQR 1-6); with natural hazards having the highest median deaths (6 [IQR 2-18]).

Conclusion

In Rwanda, MCIs have decreased, although landslides/floods are increasing, preventing a decrease in trauma-related mortality. By training journalists in “mass-casualty reporting”, the

potential of the “systematic media review” could be further enhanced, as a way to collect MCI data in settings without databases.

Introduction

Surge capacity is defined as “the ability of a healthcare system to expand beyond its regular operations and accommodate a greater number of patients in response to multiple casualty-producing events”. (1) Mass-casualty incidents (MCIs), such as those caused by road traffic accidents or natural calamities, can lead to sudden increases of patients seeking urgent care, challenging the capacity of hospitals to deliver adequate care, particularly where the system is already weakened by restricted resources and limited workforce, as in many low- and middle-income countries (LMICs) (2).

Surge capacity is facilitated by the existence of a strong health system, but can also be further developed by designing mass-casualty protocols that guide the reorganization of healthcare delivery, for example by pausing elective surgery (3), discharging patients to lower levels of care (4), calling in additional staff, or transitioning to modified triage systems (5). However, the type of response depends on multiple factors, such as local health care organization, trauma mechanism (1), and availability of resources. Contextualized knowledge of the causes and extent of MCIs in a specific health system is necessary to develop appropriate strategies to build surge capacity.

Globally, analysis of MCI epidemiology has been restricted by the complexity of linking trauma patients to the mass-trauma event, and the lack of pre-hospital data collection about the trauma event or the number of casualties involved, hindering identification of MCIs amongst all hospital trauma cases. In settings with limited resources, challenges associated with data collection are further complicated by the fact that data is often recorded in logbooks, where incomplete or low-quality data is frequent (6). To address these issues, “patient tracking”

solutions to triage and localize patients in real-time to specific MCIs and geographical areas has been proposed, although, this has not yet been systematically implemented (7,8). In high-income countries (HICs), surge capacity is frequently monitored and MCI protocols are developed using data collected in local databases (9–12). However, these protocols have limited applicability in LMICs due to vast differences in preconditions, such as the disproportion of the global physician density, which is 0.14/1000 people in Rwanda, as compared to 4.25/1000 in Germany or 2.61/1000 in the U.S (13).

Rwanda is a small but densely populated low-income nation in East Africa, with hilly topography and high annual precipitations occurring primarily in two annual "rainy seasons". The Ministry of Emergency Management compiles yearly reports on disasters (14) and provides detailed national contingency plans (15–17). Since 2011, an injury registry has been implemented at the two university teaching hospitals (18), however, a holistic understanding of MCI epidemiology is hindered by data being limited to certain types of disasters and lack of information on MCIs recorded at tertiary, provincial, or district hospitals.

These obstacles led us to look for new, feasible ways to estimate the burden of MCI in limited-resource settings. This study, therefore, aims to pilot the novel methodology "systematic media review" in Rwanda, to identify mass-trauma events and assess their epidemiology using media as the data source.

Methods

Defining mass-trauma / mass-casualty incidents

While "disaster" in the context of health care describes situations where available resources are not enough to provide adequate patient care (19), "mass-trauma" and "mass-casualty incidents" lack globally accepted definitions. Algorithms have been proposed to quantify the

maximum number of casualties that a hospital can manage at the same time, such as the Hospital Acute Care Surge Capacity (HACSC) (20) but, have not been validated in more resource-limited contexts. In this study, we defined MCIs as events causing three or more injuries, as this was expected to be the lower threshold for when health facilities in Rwanda may need to utilize "surge capacity" to meet the patient surge.

Search strategy

Data was collected through NexisUni (21), a database for non-academic publications, commonly used in sociological and medicolegal research (22,23). The search strategy was developed in English with the help of an information specialist from the Harvard Medical School Countway Library (Appendix 1) and was translated to French and Kinyarwanda. Inclusion criteria were news articles, radio/news transcripts, and governmental/non-governmental reports published between January 1st, 2010 and September 1st, 2020, which reported traumatic events that occurred in Rwanda and caused three or more injuries or, in the case of missing injury data, where the reported number of on-site deaths ≥1 and the trauma mechanism suggested the possibility of three or more injuries.

The exclusion criteria were languages other than English, French, or Kinyarwanda; occurrences before January 1st, 2010 or after September 1st, 2020; explicit mention of the total number of injuries or deaths being less than three; non-traumatic mass-casualty events; location of MCI solely outside of Rwanda; no mention of a specific event (e.g. descriptions of annual trends) or no mention of time and location. Articles were split by two study members (LV and MD) who completed eligibility screening, and data were extracted through RedCap (Appendix 2).

Data analysis

In cases where different articles described an MCI with the same trauma mechanism, province, and date (+/- one day), this was considered as the same event, unless there was information that indicated these were distinct MCIs. Articles describing the same MCI were merged and data was cleaned as following: in cases of discrepancies regarding the number of injuries, the largest number was chosen to avoid missing any data or on-site deaths; in case of discrepancies in terms of which districts were affected, the largest number of districts was chosen. Epidemiological patterns including the number of MCIs, number of injuries and on-site deaths, and temporal and geographical trends in MCI incidence, type, and number of injuries and deaths were analyzed using descriptive statistics and Fisher's exact test using Stata v16.0 (College Station, TX: StataCorp LP). P-values <0.05 were considered statistically significant.

Results

The search strategy identified 3187 articles (3026 in English, 161 in French, and none in Kinyarwanda), of which 247 met inclusion criteria (Appendix 3) and were included in the study. The most common source was The New Times (95/247, 38.5%), which is Rwanda's leading newspaper. The 247 articles included in this study described 117 mass-casualty incidents, MCIs (Appendix 4). For most trauma events, data on the number of injuries (n=99, 84.6%), on-site deaths (n=114, 97.4%) and the district(s) where the trauma occurred (n=113, 96.6%) was provided. In 60.0% (n=67) of the MCIs, data was available regarding which hospitals the patients were taken to: with the most common referral being to tertiary hospitals (n=26, 38.8%), followed by a combination of tertiary, provincial, and/or district hospitals (n=20, 29.9%), and district hospitals (n=14, 20.9%). Of the 67 events with available information, patients injured during the same event were taken to more than one hospital in 43.3% of cases (n=29). Demographic data were reported in 43 events (37.4%), of which 32 (27.4%) reported the name of ≥1 victim and 13 (11.3%) reported the age of ≥1 victim. Due to lack of consistent reporting, demographic data were excluded from further analysis.

Characteristics of mass-casualty incidents

Of 117 MCIs, 73 (62.4%) were caused by road-traffic accidents, 23 (19.7%) by natural hazards, 20 (17.1%) acts of violence/terrorism, and 1 (0.09%) by a boat collision. The total incidence of MCIs, and the number of road-traffic accidents and acts of violence/terrorism-related events, decreased over time, although the number of natural hazard events increased, making it the most common mechanism of mass-trauma between 2017-2020 (Fig 1).

For road traffic accidents (n=73), almost equal proportions were due to derailment of the vehicle off the road / overturned (n=35, 47.9%) and collisions (n=34, 46.6%). The vehicles most frequently involved were bus/cars (n=58, 79.5%), trucks (n=29, 39.7%), motorcycles (n=5, 6.9%) and bicycles (n=3, 4.1%). Pedestrians were involved in 11 accidents (15.1%). For natural hazards (n=23), landslides/flooding were the most common (n=18, 78.3%), followed by earthquakes (n=2, 8.7%), storms (n=2, 8.7%), and lightning (n=1, 4.3%). For acts of violence/terrorism(n=20), explosion/detonation was the most common mechanism (n=16, 80.0%), followed by firearms (n=2, 10.0%), machete/knife (n=1, 5.0%), and a combination of firearms and machete/knife (n=1, 5.0%).

Geographical distribution of events

33 (28.7%) MCIs took place in the Western province, 28 (24.3%) in the Southern province, 27 (23.5%) in Kigali, 25 (21.4%) in the Northern Province, 16 (13.7%) in the Eastern province, and one event where the province was not disclosed. Of these, 13 (11.1%) occurred over multiple provinces; all multiprovincial events were natural hazards. All but one of Rwanda's 30 districts (Nyamagabe, Southern province) had at least one MCI during the study period (Fig 2).

In Kigali, acts of violence/terrorism were the most common MCI, although reported as rare, isolated terrorist-related shootings and grenade blasts, where 75% occurred between 2011-2013, and only one act of violence/terrorism-related MCI occurred between 2017-2020. In the other regions, road-traffic accidents were most common, except for the Western province where road-traffic accidents and natural hazards were equally common (Table 1). The difference in proportions of trauma mechanisms, calculated for road-traffic accidents, natural hazards, and acts of violence/terrorism, was statistically significant for Kigali ($p<0.000$), the Western province ($p<0.000$) and the Southern province ($p=0.044$), but not for the other provinces (Northern: $p=0.462$; Eastern: $p=0.117$).

Injuries and deaths

The median number of injured persons per event was 11 (IQR 5-18), and the median number of on-site deaths was 2 (IQR 1-6). These numbers varied depending on the trauma mechanism, with natural hazards having the highest median deaths (6 [IQR 2-18]), followed by a boat-accident (categorized as "other") (5 [IQR 5-5]), and road-traffic accidents (2.5 [IQR 1-6]). The boat-accident had the highest median injuries (37 [IQR 37-37]), followed by natural hazards (16.5 [IQR 6-22]), and road-traffic accidents (10.5 [IQR 5-15]). The number of deaths showed a downward trend from 2010-2017 but spiked in 2018 (Fig 3), when lightning struck a crowded church, injuring 140 persons.

Discussion

The "systematic media review" is a novel methodology to assess mass-trauma epidemiology in absence of systematic data collection. Although commonly used in medicolegal research and used in one 2009 local case-series analysis of the Maryland health services (24), this is, to our knowledge, the first time that NexisUni is used for epidemiological research. Difficulties in developing global estimates of disease burden and epidemiological patterns are well-

known, with most information being derived from UN data or academic institutions in the Global North (25). For settings with limited resources and data collection, we propose the systematic media review as an opportunity to fill the global knowledge gap on mass-trauma events, which may help inform policy and clinical decision-making.

To our knowledge, there are no longitudinal studies describing the epidemiology of mass-trauma on a global scale. However, traumatic injury, measured by mortality and disability-adjusted life years, is decreasing in Rwanda and globally (26,27). In this study, we similarly found that mass-trauma events and the associated injuries have decreased in Rwanda during the past 10 years. Yet, mortality in mass-trauma events did not improve during the study period, which may be explained by the relative increase in natural hazards that tend to be associated with high victim tolls (28,29). Increased frequency and intensity of natural hazards is a known effect of climate change (43,44). Countries with weak health infrastructure are especially vulnerable (34) and it is therefore imperative that developing trauma systems and national surgical / disaster plans also take into consideration changing meteorologic patterns (45,46). According to recent data by the Ministry in Charge of Emergency Management, landslides and floods are the predominant types of natural hazards in Rwanda (14,15), when excluding lightning which is not typically included in the four main categories of natural disasters: hydrological, meteorological, climatological, and geophysical disasters (30). In our study, natural hazards, including lightning, was also the trauma mechanism with the largest number of on-site deaths, and the second highest median number of people injured, when excluding the single boat-accident categorized as “other”. Although there is limited data on the direct impact of floods (31), floods lead to increased injury and mortality compared to landslides (32,33), which aligns with our findings. In addition to the consequences on morbidity and mortality, landslides and floods cause large socioeconomic costs, as houses and workplaces may be destroyed, but these aspects were outside the aims of this study (34).

Road-traffic accidents accounted for the bulk of mass-trauma in Rwanda until 2017, which resembles patterns reported by high-income countries (9,35) and the neighboring country Tanzania (36). Globally, road-traffic accidents cause approximately 1.35 million deaths annually and between 20-50 million non-fatal injuries, with 93% of fatalities coming from LMICs (37). The African Region counts 40% more road deaths per 100,000 people compared to other LMICs, and according to the World Health Organization, most injuries and deaths involve vulnerable road users, such as pedestrians, cyclists, and motorcyclists (37). This contrasts with our findings where car and bus passengers were most affected, and pedestrians and bicyclists were involved in 15.1% and 4.1% of MCIs, respectively. This is lower than a study from Kigali where pedestrians were involved in 35% of accidents in 2012-2016 (38), although differences between Kigali City and national data may be explained by differences in urban-rural environments. The sharp decrease of road traffic accidents may be explained by the national road safety initiative, launched in 2004, where new laws were introduced to penalize excessive speed and drunk driving, and campaigns to raise awareness regarding road safety, and mandating speed governors on all public buses (39). Resultingly, casualties due to road traffic accidents decreased by 30%, and Rwanda has been championed as a successful example in improving road safety (39).

Our results showed a geographical skew of events, with natural hazards significantly more common in the Western province which is more mountainous and bordering lake Kivu, and road-traffic accidents significantly more frequent in the Southern province and Kigali. The increased vulnerability of the Western province matches the high-risk districts for natural disasters identified in the Rwandan National Contingency Plan on Floods and Landslides (15). This geospatial pattern differs from the U.S. where the mass-trauma burden is predominantly concentrated in urban centers, likely due to larger proportions of road-traffic accidents and gun violence (9). Our findings suggest that it could be appropriate in Rwanda to design sub-national strategies including prioritization of MCI protocols at provincial or district hospitals in rural areas, where over 80% of the population lives and where the most casualties caused by

natural hazards occurred in the last years (40). Additionally, referral guidelines for low-level health care facilities should be implemented, including bypass protocols to quickly enable transferring of urgent cases to higher-level facilities (41,42), to improve patient outcomes during MCIs (10). However, the hilly topography and limited accessibility to certain areas (15), particularly in the rain season, are anecdotally known as complicating factors that delay transfers of victims of landslides and floods from the trauma site to medical centers. Future strategies to manage MCIs should therefore also consider including pre-hospital care in such strained circumstances.

Finally, we propose the "systematic media review" as a method to assess MCI epidemiology in the absence of databases, utilizing a systematic search strategy and a standardized, contextually adapted data collection form modified for the media review (Appendix 5). Utilizing the systematic media review, we are introducing a new stakeholder to better understand mass-trauma epidemiology: journalists, who by profession are experts in data collection. This expertise could be further utilized by optimizing the strategy in collecting "pre-hospital data", which already occurs through news reporting, including epidemiologically relevant demographic factors, such as age or sex. In our study, national newspapers were the most common data sources, and most articles detailed in which district the trauma occurred, the number of on-site injuries, on-site deaths, and in many cases, to which hospitals the patients were transferred (60.0%). Training journalists in reporting trauma data in a systematic and standardized way could therefore be an innovative and cost-effective solution to overcome difficulties in collecting MCI data in LMICs.

Limitations

This study has multiple limitations. Firstly, the term "injuries" can range from very mild injuries not needing healthcare to severe injuries leading to death, which makes comparisons between events difficult. Similarly, the threshold for what constitutes mass-casualty incidents in Rwanda

has not been previously determined and therefore, our definition was arbitrary. A previous study of surge capacity at provincial/tertiary hospitals in the neighboring country Tanzania used a fixed number of 10 for a road traffic accident to be considered a MCI (36). Therefore, the threshold of three may be appropriate in rural areas of Rwanda, although it should possibly be adjusted upwards in urban areas.

Secondly, we don't know how many MCIs have not been reported and therefore can't state whether this method is exhaustive. When comparing our results with the number of floods, and the associated injuries and deaths as reported by the Ministry of Emergency Management (15), the numbers varied greatly (64 deaths in our study vs 124 deaths reported by the Ministry and 223 injuries in our study vs 47 injuries reported by the Ministry in July 2016-June 2018), indicating a need to train journalists in MCI reporting and validating these findings through other data sources.

Thirdly, in cases where multiple articles described the same event, there were sometimes discrepancies in the number of people injured / deaths reported. This was particularly common for natural hazards, which, unlike road traffic accidents, are not always clearly limited in time and exact location. Although some demographic data on MCI victims were provided, the relative lack of details could make it difficult to use this method to track clinical outcomes.

Conclusion

The systematic media review can be used to assess mass-trauma epidemiology in contexts where systematic data collection on MCIs is limited. In Rwanda, the number of MCIs has decreased, although landslides/floods are increasing, hindering a decrease in mortality. MCI protocols in Rwanda should put an emphasis on rural areas, where most natural hazards occurred, and include modified referral protocols for critical patients. To improve the quality of the systematic media review, there is potential in training journalists in "mass-casualty

reporting”. Further studies will pair this novel method with clinical data collection to validate the method and to give a more granular understanding of the epidemiology of MCIs in Rwanda.

Author contributions

All authors have contributed meaningfully to the conception and conduction of the study, critically reviewed and approved the paper.

References

1. Bonnett CJ, Peery BN, Cantrill SV, Pons PT, Haukoos JS, McVaney KE, et al. Surge capacity: a proposed conceptual framework. Am J Emerg Med. 2007 Mar;25(3):297–306.

2. Orach CG. Health equity: challenges in low income countries. Afr Health Sci. 2009 Oct;9 Suppl 2(Suppl 2):S49–51.

3. Soremekun OA, Zane RD, Walls A, Allen MB, Seefeld KJ, Pallin DJ. Cancellation of scheduled procedures as a mechanism to generate hospital bed surge capacity-a pilot study. Prehospital Disaster Med. 2011 Jun;26(3):224–9.

4. Kelen GD, McCarthy ML, Kraus CK, Ding R, Hsu EB, Li G, et al. Creation of surge capacity by early discharge of hospitalized patients at low risk for untoward events. Disaster Med Public Health Prep. 2009 Jun;3(2 Suppl):S10-16.

5. Nwadiaro HC, Yiltok SJ, Kidmas AT. Immediate mass casualty management in Jos University Teaching Hospital: a successful trial of Jos protocol. West Afr J Med. 2000 Sep;19(3):230–4.

6. Shivasabesan G, Mitra B, O’Reilly GM. Missing data in trauma registries: A systematic review. Injury. 2018 Sep;49(9):1641–7.

7. Tavakoli N, Yarmohammadian MH, Safdari R, Keyvanara M. Designing a model of patient tracking system for natural disaster in Iran. J Educ Health Promot. 2017;6:77.

8. Park K, Meiss K, Guerdan L, Cheng E, Burchard J, Gillis J, et al. Real-time geotracking and cataloging of mass casualty incident markers in a search and rescue training simulation: Pilot study. Am J Disaster Med. 2019 Spring;14(2):89–95.

9. Schenk E, Wijetunge G, Mann NC, Lerner EB, Longthorne A, Dawson D. Epidemiology of mass casualty incidents in the United States. Prehospital Emerg Care Off J Natl Assoc EMS Physicians Natl Assoc State EMS Dir. 2014 Sep;18(3):408–16.

10. Shartar SE, Moore BL, Wood LM. Developing a Mass Casualty Surge Capacity Protocol for Emergency Medical Services to Use for Patient Distribution. South Med J. 2017 Dec;110(12):792–5.

11. Hugelius K, Becker J, Adolfsson A. Five Challenges When Managing Mass Casualty or Disaster Situations: A Review Study. Int J Environ Res Public Health. 2020 Apr 28;17(9).

12. Marcozzi DE, Pietrobon R, Lawler JV, French MT, Mecher C, Peffer J, et al. Development of a Hospital Medical Surge Preparedness Index using a national hospital survey. Health Serv Outcomes Res Methodol. 2020 Mar 1;20(1):60–83.

13. Index Mundi. Physician density [Internet]. Index Mundi. 2020 [cited 2021 Mar 21]. Available from: https://www.indexmundi.com/g/r.aspx?v=2226

14. Republic of Rwanda Ministry in Charge of Emergency Management. Disaster Effects Situation 2018/2019. 2019.

15. Republic of Rwanda Ministry in Charge of Emergency Management. National Contingency Plan for Floods and Landslides. 2018.

16. Republic of Rwanda Ministry in Charge of Emergency Management. National Contingency Plan for Managing the Emergency Consequenecs of Terrorist Incidents. 2019.

17. Republic of Rwanda Ministry in Charge of Emergency Management. National Contingency Plan for Earthquake. 2019.

18. Petroze RT, Mumararungu A, Ndayiragije V, Ntakiyiruta G, Kyamanywa P, Reid J, et al. Patterns of Injury at Two University Teaching Hospitals in Rwanda: Baseline Injury Epidemiology Using the Rwanda Injury Registry. Rwanda Med J. 2014 Jan 1;71:5–8.

19. Roccaforte JD, Cushman JG. Disaster preparedness, triage, and surge capacity for hospital definitive care areas: optimizing outcomes when demands exceed resources. Anesthesiol Clin. 2007 Mar;25(1):161–xi.

20. Bayram JD, Zuabi S, Subbarao I. Disaster metrics: quantitative benchmarking of hospital surge capacity in trauma-related multiple casualty events. Disaster Med Public Health Prep. 2011 Jun;5(2):117–24.

21. LexisNexis Academic website [Internet]. 2021. Available from: www.nexisuni.com

22. Shih MJ, Vidovich MI. Medicolegal characteristics of aortic stenosis litigation: a review of the LexisNexis Academic database. J Heart Valve Dis. 2010 Sep;19(5):599-604: discussion 604-605.

23. Fernandez C, Fernandez A, Vidovich MI. Medicolegal Characteristics of Cardiac Tamponade Litigation: A Review of the LexisNexis Academic Database. Cardiovasc Revascularization Med Mol Interv. 2020 Jan;21(1):122–6.

24. Branas CC, Sing RF, Perron AD. A Case Series Analysis of Mass Casualty Incidents. Prehosp Emerg Care. 2000 Jan 1;4(4):299–304.

25. Byass P. The Imperfect World of Global Health Estimates. PLOS Med. 2010 Nov 30;7(11):e1001006.

26. Haagsma JA, Graetz N, Bolliger I, Naghavi M, Higashi H, Mullany EC, et al. The global burden of injury: incidence, mortality, disability-adjusted life years and time trends from the Global Burden of Disease study 2013. Inj Prev J Int Soc Child Adolesc Inj Prev. 2016 Feb;22(1):3–18.

27. James SL, Castle CD, Dingels ZV, Fox JT, Hamilton EB, Liu Z, et al. Global injury morbidity and mortality from 1990 to 2017: results from the Global Burden of Disease Study 2017. Inj Prev. 2020 Oct 1;26(Supp 1):i96.

28. Bartholdson S, von Schreeb J. Natural Disasters and Injuries: What Does a Surgeon Need to Know? Curr Trauma Rep. 2018/03/23 ed. 2018;4(2):103–8.

29. Chan EYY, Man AYT, Lam HCY. Scientific evidence on natural disasters and health emergency and disaster risk management in Asian rural-based area. Br Med Bull. 2019 Mar 1;129(1):91–105.

30. Guha-Sapir D, Hoyois P, Below R. Annual disaster statistical review 2015 [Internet]. Centre for Research on the Epidemiology of Disasters; 2016 [cited 2021 Apr 14]. Available from: http://www.cred.be/sites/default/files/ADSR_2015.pdf.

31. Saulnier DD, Brolin Ribacke K, von Schreeb J. No Calm After the Storm: A Systematic Review of Human Health Following Flood and Storm Disasters. Prehospital Disaster Med. 2017 Oct;32(5):568–79.

32. Agrawal S, Gorokhovich Y, Doocy S. Risk factors for mortality in landslide- and flood-affected populations in Uganda. Am J Disaster Med. 2013 Spring;8(2):113–22.

33. Agrawal S, Gopalakrishnan T, Gorokhovich Y, Doocy S. Risk factors for injuries in landslide- and flood-affected populations in Uganda. Prehospital Disaster Med. 2013 Aug;28(4):314–21.

34. World Health Organization. Climate change and health [Internet]. World Health Organization Newsroom. 2018. Available from: https://www.who.int/news-room/fact-sheets/detail/climate-change-and-health

35. Park JO, Shin SD, Song KJ, Hong KJ, Kim J. Epidemiology of Emergency Medical Services-Assessed Mass Casualty Incidents according to Causes. J Korean Med Sci. 2016 Mar;31(3):449–56.

36. Koka PM, Sawe HR, Mbaya KR, Kilindimo SS, Mfinanga JA, Mwafongo VG, et al. Disaster preparedness and response capacity of regional hospitals in Tanzania: a descriptive cross-sectional study. BMC Health Serv Res. 2018 Nov 6;18(1):835.

37. World Health Organization. Road traffic injuries [Internet]. World Health Organization. 2021 [cited 2021 Mar 21]. Available from: https://www.who.int/health-topics/road-safety#tab=tab_1

38. Rosenberg A, Mukeshimana L, Uwamahoro A, Dworkin M, Nsengimana V, Kankindi E, et al. The Initial Prehospital Management of Traumatic Brain Injuries in Kigali, Rwanda. Prehospital Disaster Med. 2020 Oct;35(5):533–7.

39. Brown H. Rwanda's road-safety transformation [Internet]. Bulletin of the World Health Organization. 2007 [cited 2021 Mar 21]. Available from: https://www.who.int/bulletin/volumes/85/6/07-010607/en/

40. Trading Economics. Rwanda - Rural Population [Internet]. Trading Economics. 2021 [cited 2021 Mar 21]. Available from: https://tradingeconomics.com/rwanda/rural-population-percent-of-total-population-wb-data.html

41. Odland M, Whitaker J, Nepogodiev D, Aling' C, Bagahirwa I, Dushime T, et al. Identifying, Prioritizing and Visually Mapping Barriers to Injury Care in Rwanda: A Multi-disciplinary Stakeholder Exercise. World J Surg. 2020 May 21;44.

42. Mpirimbanyi C, Abahuje E, Hirwa AD, Gasakure M, Rwagahirima E, Niyonzima C, et al. Defining the Three Delays in Referral of Surgical Emergencies from District Hospitals to University Teaching Hospital of Kigali, Rwanda. World J Surg. 2019 Aug;43(8):1871–9.

43. [Masson-Delmotte, V., P. Zhai, H.-O. Pörtner, D. Roberts, J. Skea, P.R. Shukla, A. Pirani, W. Moufouma-Okia, C. Péan, R. Pidcock, S. Connors, J.B.R. Matthews, Y. Chen, X. Zhou, M.I. Gomis, E. Lonnoy, T. Maycock, M. Tignor, and T. Waterfield (eds.)]. IPCC, 2018: Summary for Policymakers. In: Global Warming of 1.5°C. An IPCC Special Report on the impacts of global warming of 1.5°C above pre-industrial levels and related global greenhouse gas emission pathways, in the context of strengthening the global response to the threat of climate change, sustainable development, and efforts to eradicate poverty. World Meteorol Organ Geneva Switz. :32 pp.

44. Haque U, da Silva PF, Devoli G, Pilz J, Zhao B, Khaloua A, et al. The human cost of global warming: Deadly landslides and their triggers (1995-2014). Sci Total Environ. 2019 Sep 10;682:673–84.

45. Roa L, Velin L, Tudravu J, McClain CD, Bernstein A, Meara JG. Climate change: challenges and opportunities to scale up surgical, obstetric, and anaesthesia care globally. Lancet Planet Health. 2020;4(11):e538–43.

46. Pyda J, Patterson RH, Caddell L, Wurdeman T, Koch R, Polatty D, et al. Towards resilient health systems: opportunities to align surgical and disaster planning. BMJ Glob Health. 2019;4(3):e001493.

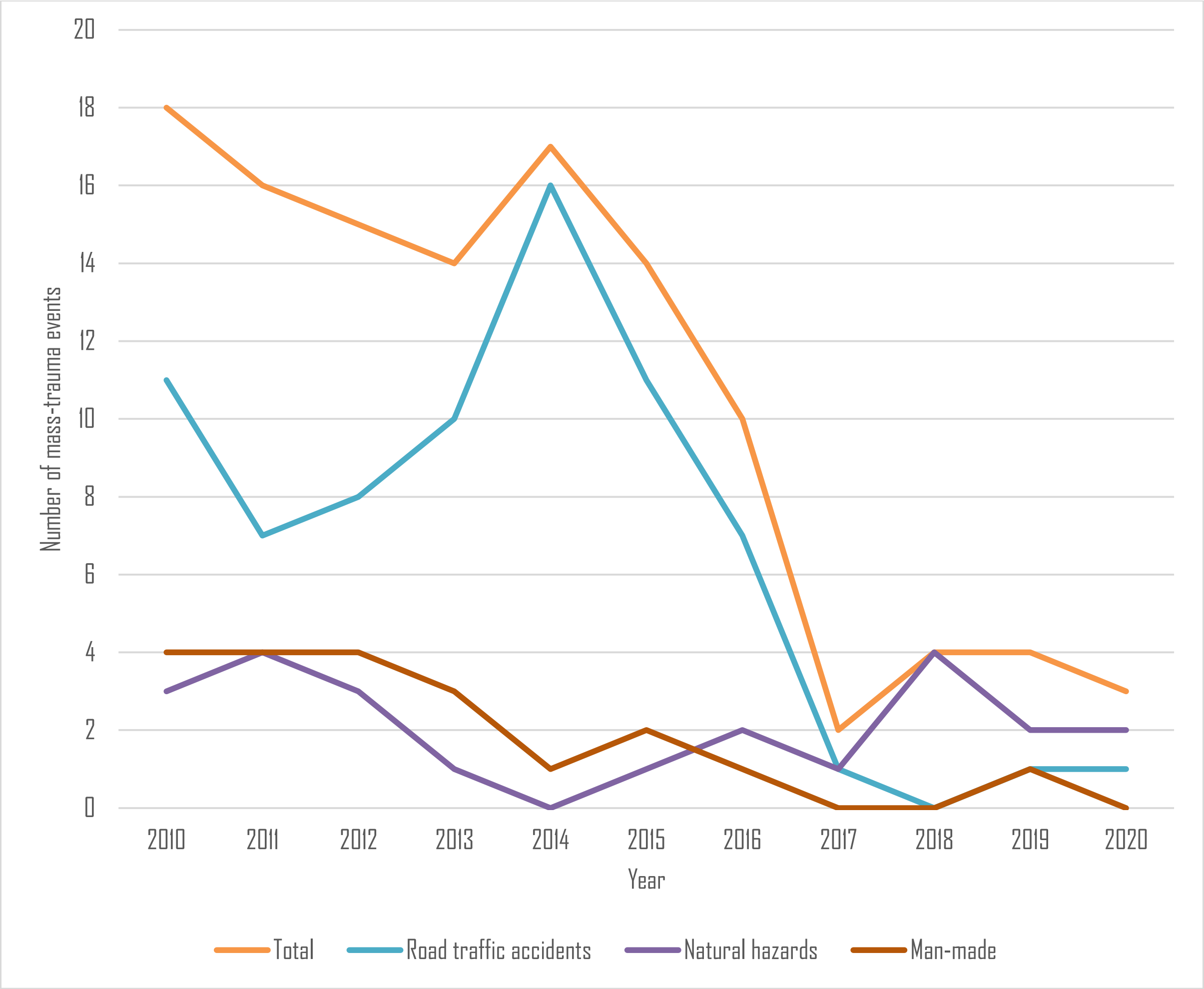

Fig 1. The number of mass-casualty incidents plotted over time, reported by media.

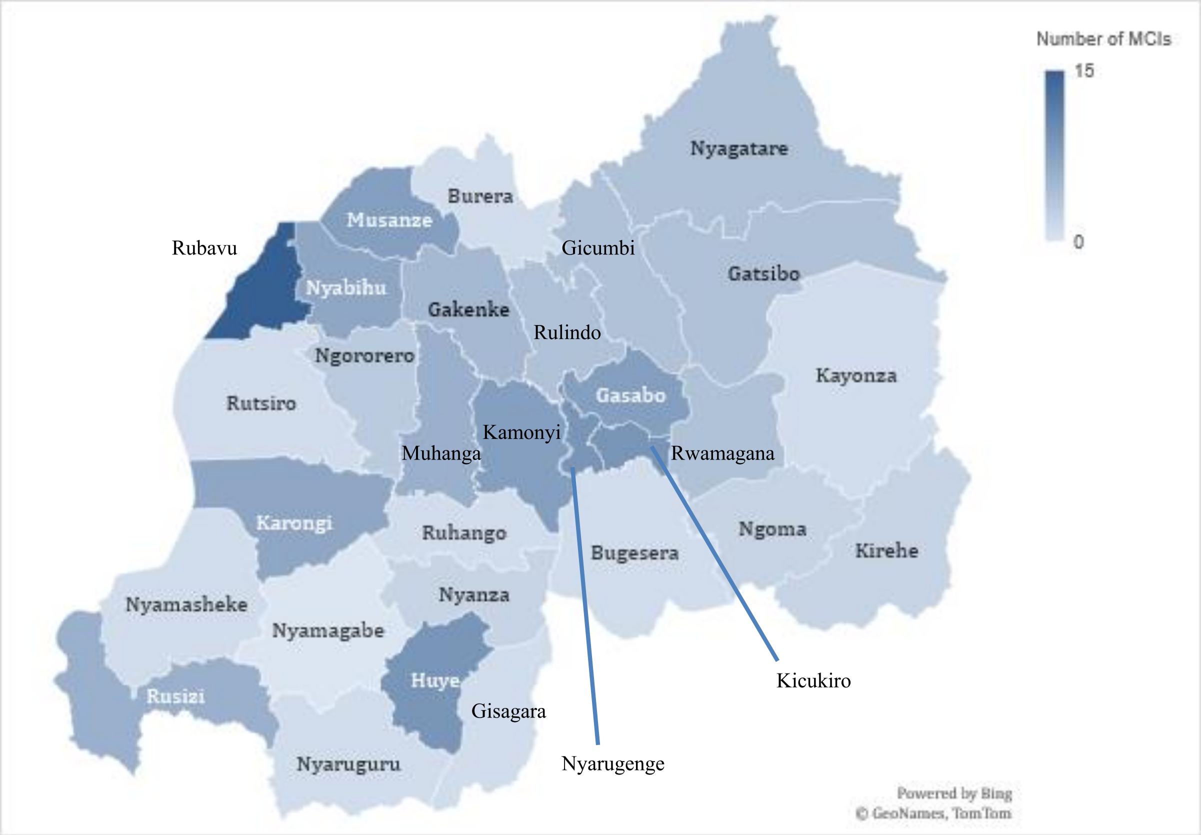

Fig 2. Geographical distribution of mass-casualty incidents in Rwanda. Map showing Rwanda's districts with the depth of color corresponding to the number of MCIs (mass-casualty incidents) reported by media between January 1st, 2010 and September 1st, 2020.

	**All MCIs, n (%)**	**Causes of MCIs, n (%)**			
		**Road-traffic accidents**	**Natural hazards**	**Acts of violence/terrorism**	**Other (boat accident)**
**Total***	117	73 (62.4)	23 (19.7)	20 (17.1)	1 (0.1)
**Kigali**	27	12 (44.4)	2 (7.4)	13 (48.1)	0 (0)
**Northern**	25	15 (60.0)	7 (28.0)	3 (12.0)	0 (0)
**Western**	33	15 (45.5)	15 (45.5)	2 (6.1)	1 (3.0)
**Southern**	28	22 (78.6)	5 (17.9)	1 (3.6)	0 (0)
**Eastern**	16	9 (56.0)	6 (37.5)	1 (6.3)	0 (0)
**Province not mentioned**	1	0 (0)	1 (100)	0 (0)	0 (0)

Table 1. Regional distribution of mass-casualty incidents by sub-type of incident. Distribution of mass-casualty incidents (MCIs) reported by media, according to geographical

province and trauma mechanism. *As some events were multi-provincial the vertical sum may exceed the total number of events.

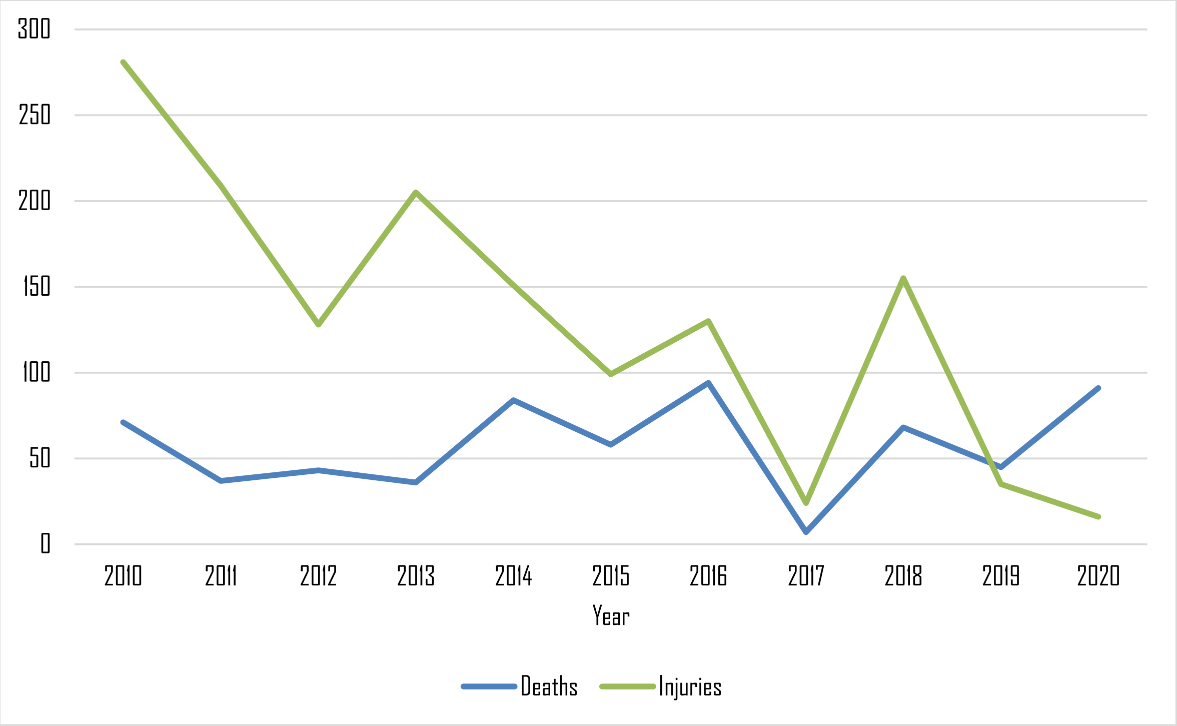

Fig 3. The number of on-site deaths and injuries due to mass-trauma per year, as reported by media.

Appendix 1. NexisUni Search strategy

Date Range: Jan 1, 2010 – September 1, 2020

ENGLISH

hlead(Rwanda) and hlead((accident*)) and hlead((road* OR traffic* OR vehicle* OR car* OR moto* OR motorcycle* OR bus* OR truck*)) and hlead((dead OR death* OR fatal* OR injur* OR wound* OR trauma OR kill*))
398 unique articles

hlead(Rwanda) and hlead((flood* OR landslide* OR earthquake* OR cyclone* OR storm* OR hurricane* OR natural hazard* OR natural disaster*)) and hlead((dead OR death* OR fatal* OR injur* OR wound* OR trauma OR kill*))
707 unique articles

hlead(Rwanda) and hlead((shoot* OR machete* OR stab* OR explosion* OR detonation* OR weapon*)) and hlead((dead OR death* OR fatal* OR injur* OR wound* OR trauma OR kill*))
1,921 unique articles

Total number of articles: 3,026

FRENCH

hlead(Rwanda)AND hlead((accident* de la circulation OR accident* de la route OR accident* de moto* OR accident* de bus OR accident* de voiture OR accident* automobile* OR accident* de camion*)) AND hlead((blessure* OR blesse* OR traumatisme* OR mort*))
23 unique articles

hlead(Rwanda) AND hlead((glissement de terrain OR eboulement* OR inundation* OR tremblement de terre OR seisme* OR cyclone* OR ouragan* OR tempete* OR catastrophe* naturelle* OR désastre*)) AND hlead((blessure* OR blesse* OR traumatisme* OR mort*))
31 unique articles

hlead(Rwanda) AND hlead((blesse* par balle OR arme* à feu* OR arme* blanche* OR machette* OR explosion* OR detonation*)) AND hlead((blessure* OR blesse* OR traumatisme* OR mort*))
107 unique articles

Total number of articles: 161

KINYARWANDA

hlead(Rwanda) and hlead(impanuka) and hlead((impanuka zo mumuhanda OR imodoka OR moto OR bus OR amakamyo)) and hlead((urupfu OR ibikomere bitera urupfu OR impanuka OR gupfa))
0 unique articles

Rwanda AND (imyuzure OR isuri OR imitingito OR serwakira OR inkubi y'umuyaga OR Ibiza) AND (urupfu OR ibikomere bitera urupfu OR impanuka OR gupfa)
0 unique articles

Rwanda AND (kurasa OR umupanga OR gusogota OR guturika OR intwaro) AND (urupfu OR ibikomere bitera urupfu OR impanuka OR gupfa)
0 unique articles

Appendix 2. REDCap Data Extraction Form

- **Article title:** __________
- **Who published article?** (newspaper name) __________
- **Language of article:** English / French / Kinyarwanda / Other
- **Date of publication (YYMMDD):** __________
- **Date of trauma event (YYMMDD):** __________
- **Geographical location of the event (province):** Northern / Western / Southern / Eastern / Kigali / Not mentioned or other
- **In which district did it occur?** Gasabo (Kigali) / Kicurkiro (Kigali) / Nyarugenge (Kigali) / Burera (Northern) / Gakenke (Northern) / Gicumbi (Northern) / Musanze (Northern) / Rulindo (Northern) / Gisagara (Southern) / Huye (Southern) / Kamonyi

(Southern) / Muhanga (Southern) / Nyamagabe (Southern) / Nyanza (Southern) / Nyaruguru (Southern) / Ruhango (Southern) / Bugesera (Eastern) / Gatsibo (Eastern) / Kayonza (Eastern) / Kirehe (Eastern) / Ngoma (Eastern) / Nyagatare (Eastern) / Rwamagana (Eastern) / Karongi (Western) / Ngororero (Western) / Nyabihu (Western) / Nyamasheke (Western) / Rubavu (Western) / Rusizi (Western) / Rutsiro (Western) / Other or unknown

- **What was the mechanism of trauma?** Road traffic accident / natural hazards / acts of violence/terroris / other
    - If "road traffic accident":
        - **What type of vehicles/persons were involved in the road traffic accident?** Bus / motorcycle / car / truck / bicycle / pedestrians / other
        - **What happened in the accident?** Collision / derailment (slid off the road) / Other
    - If "natural hazard":
        - **What type of natural hazard was it?** Landslide or flooding / earthquake / storm, hurricane or cyclone / other
    - If "acts of violence/terrorism":
        - **What type of acts of violence/terrorism-related mechanism was it?** Machete or knife / gun, rifle or firearm / explosion or detonation / other
- **Number of persons injured (not dead): __________**
- **Number of on-site deaths: __________**
- **Any other relevant information shared: __________**

Appendix 3. Included articles

Article title	Media source	Year of publication
Focus On Africa - 5:30 PM GMT	TVeyes - BBC World	2019
Rwanda; Two Children Die in Grenade Explosion	The New Times (Kigali)	2010
Rwanda; Accident Claims Three Road Workers	The New Times (Kigali)	2010
Rwanda; One Dead, 24 Injured in Road Accident	The New Times (Kigali)	2010
Grenade blast wounds 16 in Rwandan capital: police	Agence France Press	2010
Grenade explodes in western Rwanda as two other attacks hit capital 4	BBC Monitoring Africa - Political	2010
Un mort et 16 blessés dans une série d'eplosions à Kigali	Xinhua News Agency - French	2010
Rwanda: Floods kill four in western district of Rubavu	BBC	2010
Rwanda: Floods Kills Four in Western Province	The New Times	2010
Rwanda: Twenty feared dead as boat capsizes in Lake Kivu	BBC	2010
Rwanda; 20 Mourners Feared Dead as Boat Capsizes in L. Kivu	The New Times	2010

Rwanda; Five Seriously Injured in Kanogo Accident	The New Times (Kigali)	2010
Rwanda; 40 Injured in Two Separate Road Accidents	The New Times (Kigali)	2010
Rwanda; 40 Injured in Two Separate Road Accidents	The New Times (Kigali)	2010
Rwanda; 12 Injured in Road Accident	The New Times (Kigali)	2010
Rwanda; 12 Injured in Road Accident	The New Times (Kigali)	2010
Pair of grenade attacks kills 2 and hurts 32 in Rwanda; Explosions rock capital as country prepares for presidential election	The International Herald Tribune	2010
Rwanda; One Dead, 28 Wounded in Grenade Attacks	The New Times (Kigali)	2010
Rwanda Blast Critically Wounds 7	SmarTrend	2010
Rwanda; Grenade Victim Dies	The New Times (Kigali)	2010
Rwanda: explosion d'une grenade à Kigali, au moins sept blessés (police)	Agence France Press	2010
Rwanda: explosion d'une grenade à Kigali, au moins sept blessés (police)	La Tribune de Geneve.fr	2010
Rwanda : explsion d'une grenade à Kigali (presse)	Xinhua News Agency - French	2010
Point de l'actualité à 21H00 GMT	Xinhua News Agency - French	2010
Rwanda grenade attack marks Kagame's presidential win	Agence France Press - English	2010
AF-Rwanda-Election, Update	The Canadian Press	2010
Rwanda vote terror	The Advertiser (Australia)	2010
Rwanda; 25 Injured in Road Accidents in Rwamagana District	The New Times (Kigali)	2010
Rwanda; Seven Injured in City Road Accident	The New Times (Kigali)	2010
Rwanda; Three Dead, 13 Injured in Road Accident	The New Times (Kigali)	2010
Rwanda: Six Miners Killed by Landslide	The New Times	2010
wanda; Canadian Family Survives Fatal Accident	The New Times (Kigali)	2011
Seven injured in grenade explosion in Rwanda	BBC Monitoring Africa - Political	2011
Two killed in Rwanda grenade explosion	Agence France Press	2011
Two killed, 26 injured in grenade explosion in Rwandan capital	BBC Monitoring Africa - Political	2011
Rwanda: deux morts et 26 blessés dans l'explosion d'une grenade à Kigali	Agence France Press	2011
L'explosion d'une grenade fait deux morts près de Kigali, au Rwanda	The Canadian Press	2011
Rwanda; One Dies in Bus Accident	The New Times (Kigali)	2011
10 injured in grenade explosion in Rwanda	Xinhua General News Service	2011

Rwanda : une explosion à la grenade fait 10 blessés	Xinhua News Agency - French	2011
5 people dead in Rwanda boat collision	Xinhua General News Service	2011
Rwanda; Over 30 Injured in Bus Accident	The New Times (Kigali)	2011
Rwanda; 11 Perish in Road Accident	The New Times (Kigali)	2011
Rwanda; Five Injured in Accident	The New Times (Kigali)	2011
Grenade explosion injures 21 in western Rwanda	BBC Monitoring Africa - Political	2011
Rwanda; Grenade Blast Injures 21 in Rusizi	The New Times (Kigali)	2011
Grenade blast wounds 21 in Rwanda	Agence France Press	2011
Au Rwanda, plusieurs dizaines de blessés après un attentat à la grenade...	Renseignor	2011
Rwanda; 15 Injured in Commuter Taxi Accident	The New Times (Kigali)	2011
Rwanda; Landslide in Nyabihu Kills Three Teenagers	The New Times	2011
Rwanda; Five Injured in Accident	The New Times (Kigali)	2011
Rwanda: Lighting kills student as floods destroy 12 homes	The New Times	2011
Rwanda; Inatek Teacher Killed in Car Accident	The New Times (Kigali)	2011
Rwanda: 10 Killed in Karongi Floods	The New Times	2011
Rwanda; Children Who Perished in Landslide Laid to Rest	The New Times	2011
Rwanda; Two Killed in Rulindo Accident	The New Times (Kigali)	2011
Rwanda; Grenade Explosion Kills Two	The New Times (Kigali)	2012
Rwanda: deux morts dans l'explosion d'une grenade à Kigali (police)	Agence France Press	2012
Nouvelle attaque à la grenade à Kigali	Radio France Internationale	2012
Grenade blast in Rwanda was criminal act: police	Agence France Press	2012
Dix blessés dans l'explosion d'une grenade dans le centre du Rwanda	Agence France Press	2012
Rwanda; Three Survive Fatal Road Accident	The New Times (Kigali)	2012
Rwanda; Six Injured in Road Accident	The New Times (Kigali)	2012
Rwanda; Eight Killed in Bus Accident	The New Times (Kigali)	2012
Explosion in Rwanda leaves one dead, five injured:police	Agence France Press	2012
Rwanda: un mort et 5 blessés dans une explosion dans le nord (police)	Agence France Press	2012
Rwanda: six blessés par deux grenades dans Kigali et sa banlieue (police)	Agence France Press	2012
Rwanda: Flood Death Toll Rises	The New Times	2012

Rwanda; Floods Kill Two in Musanze, Nyabihu	The New Times	2012
Rwanda floods kill 8 people	Xinhua General News Service	2012
In Brief: Floods displace hundreds in Rwanda	IRIN Africa Service	2012
Rwanda; Floods Kill, Displace Many in Nation	East African Business Week	2012
Rwanda; Two Killed, 11 Injured in Road Accident	The New Times (Kigali)	2012
Rwanda; Truck Rams Into House, Kills 8	The New Times (Kigali)	2012
Rwanda; Eight Killed in Fatal Car Accident	The New Times (Kigali)	2012
Rwanda; Five Perish in Rubavu Road Accident	The New Times (Kigali)	2012
Rwanda; Heavy Rains Kill One, Destroy Property	The New Times	2012
Rwanda; One Dead, Six Injured in City Accident	The New Times (Kigali)	2012
Rwanda; Pupil Killed in Road Accident	The New Times (Kigali)	2012
Rwanda; Kamonyi Accident Claims Two	The New Times (Kigali)	2012
Rwanda; Four Policemen Killed in Nyanza Road Accident	The New Times (Kigali)	2013
Rwanda; Nyanza Accident Kills Four Policemen	The New Times (Kigali)	2013
Rwanda; Musanze Accident Claims One, Injures Six	The New Times (Kigali)	2013
Rwanda; Gloomy Trip. Nur Students Injured in Road Accident	The New Times (Kigali)	2013
Rwanda; Nur Students Injured in Road Accident	The New Times (Kigali)	2013
Nur Students Injured in Road Accident	The New Times (Kigali)	2013
Rwanda; Grenade Blast Kills One	The New Times (Kigali)	2013
Rwanda; Two Arrested Over Grenade Explosion	The New Times (Kigali)	2013
Rwanda: Un tué, huit blessés dans un attentat à la grenade à Kigali (police)	Agence France Press	2013
Rwanda - Rwanda : attentat meurtrier à Kigali	RFI	2013
Rwanda: attentat meurtrier à Kigali	Le Figaro Newsflash	2013
Rwanda; Six Killed, Several Injured in Butare Road Accident	The New Times (Kigali)	2013
Rwanda; Six Die in Huye Road Accident	The New Times (Kigali)	2013
Rwanda; 50 Survive Road Accident	The New Times (Kigali)	2013
Rwanda; Six Dead, 16 Injured in Kirehe Road Accident	The New Times (Kigali)	2013
Rwanda; Six Dead, 16 Injured in Kirehe Road Accident	The New Times (Kigali)	2013
Rwanda; Six People Killed in a Accident	The New Times (Kigali)	2013

Three arrested following fatal grenade attack	Economist Intelligence Unit (EIU) Country Reports	2013
Grenade blast in Rwanda capital kills 2 people, leaves others seriously wounded, police say; Police: 2 people dead in grenade blast in Rwanda	Canadian Press	2013
Police: 2 people dead in grenade blast in Rwand	The New Zealand Herald	2013
Rwanda; Two Dead, Eight Critically Injured in Rusizi Accident	The New Times (Kigali)	2013
Rwanda; Two Die, Eight Hurt in Rusizi Road Accident	The New Times (Kigali)	2013
Rwanda; Heavy Rains Kill Two Children in Nyagatare	The New Times	2013
Explosive Start to Rwandan Parliamentary Election Polling Mars Kagame's Victory	ThinkAfricaPress	2013
Rwanda: deux personnes tuées par des grenades avant les législatives (police)	Agence France Press	2013
Rwanda : La campagne pour les législatives perturbée par de nombreux incidents	Les Echos	2013
Rwanda : La campagne pour les législatives perturbée par de nombreux incidents	Les Echos	2013
Explosive Start to Rwandan Parliamentary Election	ThinkAfricaPress	2013
Rwanda: Un mort dans l'explosion d'une grenade à Kigali à 36 h des législatives (police)	Agence France Press	2013
Attentat à la grenade dans un marché de la capitale rwandaise...	Renseignor	2013
Rwanda - Rwanda: des opposants en exil responsables d'une attaque meurtrière à Kigali?	RFI	2013
Législatives rwandaises - Coup d'envoi explosif à Kigali	L'Observateur Paalga (Ouagadougou)	2013
Rwanda; 50 Injured in Kayonza District Road Accident	The New Times (Kigali)	2013
Rwanda; Two Dead, One Injured in Rubavu Accident	The New Times (Kigali)	2014
Rwanda; 18 Injured in Musanze Road Accident	The New Times (Kigali)	2014
Rwanda; Rubavu Accident Claims One, Injures Five	The New Times (Kigali)	2014
Rwanda; 14 Injured in Road Accident	The New Times (Kigali)	2014
Rwanda; Nation Mourns Gatsibo Road Accident Victims	The New Times (Kigali)	2014
Rwanda; Who Is to Blame for the Fatal Road Accidents?	The New Times (Kigali)	2014
Rwanda; 15 Passengers Killed, 24 Injured Following Eastern Province Bus Accident	The New Times (Kigali)	2014
Over 12 die in road accident in eastern Rwanda	BBC Monitoring Africa - Political	2014

Rwanda; Kiziguro Road Accident - All Injured Now Stable	Rwanda Focus (Kigali)	2014
Rwanda; Kiziguro Road Accident - All Injured Now Stable	Rwanda Focus (Kigali)	2014
Rwanda; Bride, Groom Involved in Road Accident Out of Danger - Medics	The New Times (Kigali)	2014
Rwanda; Bride, Groom Involved in Road Accident Out of Danger - Medics	The New Times (Kigali)	2014
Rwanda; Huye Road Accident Kills Four-Year-Old, Injures 13	The New Times (Kigali)	2014
Rwanda; Weekend Accidents Claim 20	The New Times (Kigali)	2014
Rwanda; Weekend Accidents Claim 20	The New Times (Kigali)	2014
Rwanda; Murekezi Calls On Police to Do More About Road Accidents	Rwanda Focus (Kigali)	2014
Rwanda; Murekezi Calls On Police to Do More About Road Accidents	Rwanda Focus (Kigali)	2014
Une dizaine de morts et plusieurs blessés dans un accident de la route au sud du Rwanda	PANAPRESS - Pan African News Agency	2014
Rwanda; Murekezi Calls On Police to Do More About Road Accidents	Rwanda Focus (Kigali)	2014
Rwanda; Murekezi Calls On Police to Do More About Road Accidents	Rwanda Focus (Kigali)	2014
Rwanda RDF condemns soldiers shooting spree that left four people death	The New Times (Kigali)	2014
Les accidents de la route ont fait 37 morts en une semaine au Rwanda	PANAPRESS - Pan African News Agency	2014
Les accidents de la route ont fait 37 morts en une semaine au Rwanda	PANAPRESS - Pan African News Agency	2014
Rwanda; Two Killed in Christmas Morning Accident in City	The New Times (Kigali)	2014
Rwanda; Five Killed in Rulindo Bus Accident	The New Times (Kigali)	2015
Rwanda; Five Dead, Including Kigali Basketball Club Player, in Traffic Accident	Rwanda Focus (Kigali)	2015
Rwanda; Oil Tanker Crashes Into Home, Bursts into Flames	The New Times (Kigali)	2015
Rwanda; Student Killed, Five Injured in Kicukiro Accident	The New Times (Kigali)	2015
Rwanda; Seven Prisoners Die in Car Crash, Nine Others Injured	The New Times (Kigali)	2015
Rwanda; Seven Inmates Die in Karongi Accident	The New Times (Kigali)	2015
5.8-magnitude quake injures 4 in Rwanda	Xinhua General News Service	2015
Six morts et des blessés dans un accident de la route à Rusizi	Rwanda News Agency	2015
Rwanda; One Dead, Nine Injured in Burera Road Accident	The New Times (Kigali)	2015
Grenade explosion in Rwanda kills1,injuries5	Xinhua General News Service	2015
Rwanda; One Dead, 8 Injured in Rubavu Hospital Accident	The New Times (Kigali)	2015

18 killed in road accident in Rwanda	Xinhua General News Service	2015
Eighteen killed as truck crashes into minibus in Rwanda	Deutsche Presse-Agentur	2015
Rwanda; Truck Crushes Minibus, Leaves 18 People Dead	The New Times (Kigali)	2015
Eighteen killed in road accident in Rwanda	BBC Monitoring Africa - Political	2015
Rwanda; Four Die, 15 Injured in Ngororero Road Accident	The New Times (Kigali)	2015
Quatre morts et quinze blessés dans un accident de la route à Ngororero	Rwanda News Agency	2015
Huit morts dans un accident de la route au nord du Rwanda	Rwanda News Agency	2015
Une attaque à la machette fait trois blessés dans un camp de réfugiés congolais au Rwanda	PANAPRESS - Pan African News Agency	2015
Rwanda; Two Killed, Ten Injured in Kigali-Gicumbi Road Accident	The New Times (Kigali)	2016
Rwanda; APR Handball Star Shimirwa Dies in an Accident	The New Times (Kigali)	2016
Quatre morts et plusieurs blessés dans un accident de la route à Huye	Rwanda News Agency	2016
Landslides kill nearly 50 in Rwanda	Digital Journal	2016
Landslides kill nearly 50 in Rwanda	Agence France Press	2016
Official: 49 people killed in Rwanda landslides amid rains; Official: 49 people killed in Rwanda landslides amid rains	Canadian Press	2016
20 killed by Rwanda landslides	Africa Review	2016
1ST LEAD	Deutsche Presse-Agentur	2016
Death toll from heavy rains, landslides rises to 49 in Rwanda	Xinhua General News Service	2016
Heavy rains trigger landslides in Rwanda, killing 49	Deutsche Presse-Agentur	2016
20 killed, mostly children, by Rwanda landslides	Agence France Press	2016
Death toll rises to 18 in Rwanda landslides	Xinhua General News Service	2016
Death toll from heavy rains in Rwanda climbs to 72	Xinhua General News Service	2016
Dozens dead, hundreds homeless after landslides in northern Rwanda	dpa-AFX	2016
Official: 49 people killed in Rwanda landslides amid rains	The Associated Press	2016
Rwanda: Landslides kill 22	Turkish Governmental News	2016
Heavy rains trigger landslides in Rwanda, killing 49	dpa-AFX	2016
Nineteen die in Rwanda floods	BBC Monitoring Africa - Political	2016
Rwanda; Landslide Disaster Leaves 34 Dead	News of Rwanda	2016

Xinhua world news summary at 1530 GMT, May 9	Xinhua General News Service	2016
Landslides In Northern Rwanda Kill 49	International Business Times News	2016
Rwanda; Experts Weigh in On How Deadly Floods, Landslides Can Be Prevented	The New Times (Kigali)	2016
East Africa; El Nino-Induced Floods Ravage East Africa	Deutsche Welle	2016
Deadly Rwanda floods leave thousands homeless	Al Jazeera Africa	2016
25 killed in Rwanda landslides	IANS-English	2016
Dozens killed in Rwanda landslides; World news in brief	The Independent	2016
49 killed in landslides after Rwanda downpour; world in brief	The Evening Standard	2016
Rwanda; Floods Claim 49 Lives	Rwanda Focus	2016
49 die in landslides in torrential rain	The Herald	2016
Rwanda; Survivors Recount Ordeal After Fatal City Road Accident	The New Times (Kigali)	2016
Rwanda; City Accident Victims Discharged	The New Times (Kigali)	2016
Rwanda; Seven Killed, Nine Injured in City Road Accident	The New Times (Kigali)	2016
Rwanda; Scores Killed in Kicukiro Nasty Accident	Rwanda Focus (Kigali)	2016
Un camion perd ses freins et provoque un accident mortel à Kigali	Rwanda News Agency	2016
Rwanda; Weekend Accident Claims One, Injures 27 in Rubavu	The New Times (Kigali)	2016
Rwanda; Four Die, Nine Injured in City Road Accident	The New Times (Kigali)	2016
Police kill three terror suspects in western Rwanda	Xinhua General News Service	2016
Rwanda; Volcano Passenger Bus Involved in Accident on Route to Kampala	Rwanda Focus (Kigali)	2016
Rwanda; 4.8 Magnitude Earthquake Kills One, Injures Over 20	Rwanda Focus (Kigali)	2016
4.8 magnitude quake hits western Rwanda - USGS	Xinhua General News Service	2016
Rwanda;four injuries and one dead in police shootout	The New Times (Kigali)	2016
Rwandan police officer kills colleague, injures three	Xinhua General News Service	2016
Xinhua world news summary at 0030 GMT, Oct. 3	Xinhua General News Service	2016
Rwanda; Three RDF Soldiers Killed, 20 Injured in Truck Accident	The New Times (Kigali)	2016
Three RDF Soldiers Killed, 20 Injured in Truck Accident	The New Times (Kigali)	2016
Three RDF Soldiers Killed in Road Accident Laid to Rest	The New Times (Kigali)	2016

Rwanda; Three RDF Soldiers Killed in Road Accident Laid to Rest	The New Times (Kigali)	2016
Rwanda; Police Issue Warning Against Reckless Driving	The New Times (Kigali)	2017
Deadly Windly Wreak Havoc in Rwanda	CAJ News Agency	2017
Over 1,000 people displaced by floods in western Rwanda	BBC Monitoring Africa - Political	2018
'Dead bodies everywhere' 16 killed and 140 injured as freak LIGHTNING strike hits church	Daily Star Onlien	2018
Heavy rains, landslides kill 18 in Rwanda	The Sun (Nigeria)	2018
Heavy Rains, Landslides Kill 18 in Rwanda	Africa Newswire	2018
Fatal Landslides	Daily News (South Africa)	2018
Rwanda's recurring landslides worse this year with over 200 people dead	Ventures Africa	2018
At least 12 dead, several missing after landslide in Rwanda	dpa international (Englischer Dienst)	2018
Landslides kill 18 in Rwanda, death toll at 200 since January	The Daily Monitor	2018
Latest Rwandan landslides kill at least 18 people	UPI	2018
Mudslide kills 12	Daily News (South Africa)	2018
Landslides leave 18 dead	The Herald	2018
Landslides leave 18 dead	The Herald (Glasgow)	2018
Rains wreak havoc in Rwanda, 18 killed	Anadolu Agency (AA)	2018
Flood deaths; GLOBAL BRIEFING	Daily Post (North Wales)	2018
Official: 18 killed amid flooding, landslides in Rwanda	The Associated Press	2018
Landslides in Western Rwanda kill 18 People After Heavy Rains on Weekend	Sputnik News Service	2018
Rain tolls upp by 18	The Sun	2018
No Headline In Original	Midi Libre	2018
Rwanda Mourns Deaths of 14 Miners as Official Urges More Safety Steps	Voice of America News	2019
14 Killed in Landslide	The Sun	2019
18 dead in heavy floods in Rwanda	Global English	2019
Heavy Rainfall in Rwanda leaves 10 dead	Global English	2019
8 dead, 17 injured in road accident in western Rwanda	Xinhua General News Service	2019
Eight killed, 18 hurt in Rwanda attack	The Mercury (South Africa)	2019
Unknown assailants kill eight people near Rwanda's volcanoes park	Iran Daily	2019
Eight killed,18 wounded in Rwanda attack	The Daily Monitor	2019

Eight killed,18 wounded in Rwanda attack	Daily Nation (Kenya)	2019
Unknown assailants kill eight people near Rwanda's volcanoes park	Iran Daily	2019
8 killed at tourist spot	Daily Record and Sunday Mail	2019
Attaque mortelle au Rwanda	Ouest-France	2019
13 killed in Rwanda landslides	Xinhua General News Service	2020
13 killed in Rwanda landslides	Xinhua General News Service	2020
Death toll climbs to 19 in Rwanda's heavy rains	Xinhua General News Service	2020
Landslides kill 13, wound 2 in Rwanda	Global English (Middle East and North Africa Financial Network)	2020
7 killed, 8 injured in road accident in Rwanda	Xinhua General News Service	2020
At least 65 killed in flooding, landslides in Rwanda; At least 65 killed in flooding, landslides in Rwanda	Canadian Press	2020
Texas salon owner jailed for defying governor's order freed NEWS BRIEFING Staff and news services Virus hospitalization is new barrier to military enlistment White father and son arrested in shooting death of black man GOP fundraiser and political donor to be postmaster general Rwanda flooding, landslides leave at least 65 dead Google scraps smart-city development for Toronto	The Hartford Courant	2020
Texas salon owner jailed for defying governor's order freed Virus hospitalization is new barrier to military enlistment Advocates: More immigrant deaths without Trump action GOP fundraiser and political donor to be postmaster general Rwanda flooding, landslides leave at least 65 dead Google scraps smart-city development for Toronto	The Morning Call	2020
Floods kill 65 in Rwanda as heavy rains pound East Africa	Agence France Press	2020
Flooding and landslides kill at least 65 in northern Rwanda; An estimated 11,000 people have been affected by heavy rain this year and 1,000 hectares of crops have been destroyed	The Telegraph	2020
1st LD Writethru: 55 people killed as heavy rains batter Rwanda	Xinhua General News Service	2020
Urgent: 55 people killed in Rwanda heavy rains	Xinhua General News Service	2020
Rwanda: Torrential rains kill scores in Rwanda	Thai News Service	2020
55 die in Rwandan floods, mudslides	Cape Argus (South Africa)	2020

In brief	Northern Advocate (New Zealand)	2020
Flash Flooding Kills 55 Across Rwanda - Reports	Sputnik News Service	2020
More than 70 dead in Rwanda after floods, landslides	UPI	2020
At least 65 killed in Rwanda flooding, landslides	The Indian Awaaz	2020

Appendix 4. All mass-trauma events identified in the media review.

Type of injury	Province	District(s)	Number of on-site deaths	Number of people injured	Hospital(s) specified in article	Date (YY-MM-DD)	Hospital level
RTA	Southern	Huye	3	35	Yes	2010-02-12	Tertiary hospital
RTA	Southern	-	1	24	Yes	2010-02-14	Tertiary hospital, district hospital
Violence/ terrorism	Kigali	-	0	16	No	2010-03-05	-
Natural hazard	Western	Nyabihu, Rubavu	4	17	No	2010-03-26	-
Natural hazard	Western	Rutsiro	20	19	Yes	2010-04-07	Provincial hospital
RTA	Kigali	Nyarugenge	0	13	Yes	2010-04-08	Tertiary hospital
RTA	Eastern	Rwamagana	0	35	Yes	2010-05-02	Tertiary hospital, provincial hospital
RTA	Western	Karongi	0	4	Yes	2010-05-02	Health center
RTA	Northern	Gakenke	0	12	No	2010-05-06	-
RTA	Northern	-	0	12	Yes	2010-05-06	District hospital
Violence/ terrorism	Kigali	Kicukiro, Nyarugenge	2	32	Yes	2010-05-15	-
RTA	Kigali	Kicukiro	7	9	Yes	2010-06-10	Tertiary hospital
Violence/ terrorism	Kigali	Kicukiro	0	27	Yes	2010-08-11	Tertiary hospital
Violence/ terrorism	Kigali	-	-	7	No	2010-08-13	-
RTA	Eastern	Rwamagana	25	0	Yes	2010-10-09	Provincial hospital
RTA	Southern	Gisagara	3	13	Yes	2010-11-27	Tertiary hospital
Natural hazard	Eastern	Ngoma	6	-	No	2010-12-03	-

RTA	Southern	Kamonyi	0	6	Yes	2010-12-30	Tertiary hospital
Violence/ terrorism	Kigali	Nyarugenge	0	7	No	2011-01-03	Tertiary hospital
Violence/ terrorism	Kigali	Gasabo	2	28	No	2011-01-28	-
RTA	Northern	Rulindo	1	19	Yes	2011-02-17	Tertiary hospital
Violence/ terrorism	Kigali	Nyarugenge	0	10	Yes	2011-03-01	Tertiary hospital
Boat accident	Western	Rubavu	5	37	No	2011-05-19	-
RTA	Eastern	Nyagatare	0	36	Yes	2011-06-05	Tertiary hospital, District hospital
RTA	Western	Ngororero	10	7	Partly	2011-07-01	Tertiary hospital, District hospital
RTA	Northern	Rulindo	0	5	No	2011-07-11	-
Violence/ terrorism	Western	Rusizi	0	21	Yes	2011-07-12	District hospital
RTA	Kigali	Gasabo	0	15	Yes	2011-08-02	Tertiary hospital, district hospital
Natural hazard	Western	Nyabihu	3	-	Yes	2011-08-02	Health center
RTA	Southern	Huye	0	5	Yes	2011-09-09	District hospital
Natural hazard	Western	Nyabihu	1	16	Yes	2011-09-29	District hospital, Health center
Natural hazard	Western	Karongi	10	-	No	2011-11-04	-
Natural hazard	Southern	Muhanga	3	-	No	2011-11-10	-
RTA	Northern	Rulindo	2	3	Yes	2011-12-25	Tertiary hospital
Violence/ terrorism	Kigali	Gasabo	2	18	Yes	2012-01-03	Tertiary hospital, District hospital
Violence/ terrorism	Southern	Muhanga	0	10	No	2012-01-24	-
RTA	Southern	Huye	0	3	Yes	2012-01-25	Tertiary hospital
RTA	Northern	Musanze	0	6	Yes	2012-02-25	Provincial hospital

RTA	Southern	Kamonyi	8	30	Yes	2012-03-16	Tertiary hospital, District hospital
Violence/ terrorism	Northern	Musanze	1	5	No	2012-03-23	-
Violence/ terrorism	Kigali	Nyarugenge	0	6	No	2012-03-30	-
Natural hazard	Northern, Western	Nyabihu, Musanze, Rubavu	5	-	No	2012-04-11	-
Natural hazard	Northern, Western	Musanze, Rubavu	8	-	No	2012-04-16	-
RTA	Western	Rubavu	2	11	Yes	2012-05-10	District hospital
RTA	Southern	Ruhango	8	15	Yes	2012-06-27	District hospital, Health center
RTA	Western	Rubavu	5	8	No	2012-07-12	Tertiary hospital, District hospital
Natural hazard	Western	Rubavu	1	-	No	2012-09-08	-
RTA	Kigali	Kicukiro	1	6	Yes	2012-09-09	Tertiary hospital
RTA	Southern	Kamonyi	2	10	Yes	2012-12-17	Tertiary hospital
RTA	Southern	Nyanza	4	5	Yes	2013-01-04	Tertiary hospital
RTA	Northern	Musanze	1	6	Yes	2013-01-28	Provincial hospital
RTA	Southern	Kamonyi	0	15	Yes	2013-03-25	Tertiary hospital
Violence/ terrorism	Kigali	Gasabo	1	8	Yes	2013-03-26	District hospital
RTA	Southern	Huye	6	20	Yes	2013-05-03	Tertiary hospital
RTA	Southern	Nyanza	0	15	No	2013-06-05	-
RTA	Eastern	Nyagatare	5	-	No	2013-06-25	-
RTA	Eastern	Kirehe	6	16	Yes	2013-07-18	Tertiary hospital, District hospital
RTA	Western	Rusizi	5	12	No	2013-07-18	-
Violence/ terrorism	Kigali	Nyarugenge	3	32	No	2013-07-26	-

RTA	Western	Rusizi	2	8	Yes	2013-08-04	District hospital
Natural hazard	Eastern	Nyagatare	2	4	No	2013-09-01	-
Violence/ terrorism	Kigali	Kicukiro	1	14	No	2013-09-14	-
RTA	Eastern	Kayonza	-	50	Partly	2013-10-29	Tertiary hospital, District hospital
RTA	Western	Rubavu	2	1	Yes	2014-01-30	District hospital
RTA	Northern	Musanze	0	18	Yes	2014-02-12	Provincial hospital, District hospital
RTA	Western	Rubavu	1	5	Yes	2014-04-02	Tertiary hospital, District hospital
RTA	Kigali	Gasabo, Kicukiro	0	14	No	2014-04-23	Tertiary hospital, Provincial hospital
RTA	Eastern	Gatsibo	15	24	Yes	2014-07-22	Tertiary hospital, District hospital
RTA	Southern	Huye	0	4	Yes	2014-07-27	Tertiary Hospital, District hospital
RTA	Southern	Huye	1	13	Yes	2014-08-01	Tertiary
RTA	Southern	Kamonyi	12	14	Yes	2014-08-09	Tertiary Hospital
RTA	Northern	Gakenke	7	-	No	2014-08-09	-
RTA	Southern	Huye	1	3	No	2014-08-09	-
RTA	Southern	Muhanga, Kamonyi	12	13	No	2014-08-09	-
RTA	Northern	Musanze	3	-	No	2014-08-10	-
RTA	Kigali	Nyarugenge	7	10	No	2014-08-10	-
Violence/ terrorism	Northern	Gicumbi	4	7	Yes	2014-08-10	Tertiary Hospital
RTA	Northern	Gakenke	5	21	No	2014-08-10	-
RTA	Southern	Muhanga	12	-	No	2014-08-10	-
RTA	Kigali	Kicukiro	2	4	No	2014-12-25	-

RTA	Northern	Rulindo	3	18	No	2015-02-07	-
RTA	Southern	Kamonyi	5	15	Yes	2015-03-01	Tertiary hospital, District hospital
RTA	Western	Nyabihu	3	1	Yes	2015-06-25	Health center
RTA	Kigali	Kicukiro	1	5	Yes	2015-07-09	Tertiary hospital, District hospital
RTA	Western	Karongi	7	12	No	2015-07-09	Tertiary hospital, Provincial hospital
Natural hazard	Not mentioned	Not mentioned	0	4	No	2015-08-07	-
RTA	Western	Rusizi	6	-	Yes	2015-08-19	District hospital
RTA	Northern	Burera	1	9	Yes	2015-08-24	Provincial hospital
Violence/ terrorism	Kigali	Gasabo	1	5	Yes	2015-08-29	District hospital
RTA	Western	Rubavu	1	8	Yes	2015-09-03	Tertiary hospital, District hospital
RTA	Eastern	Rwamagana	18	2	Yes	2015-09-21	Tertiary hospital
RTA	Western	Ngororero	4	15	Yes	2015-10-01	Tertiary hospital
RTA	Northern	Gicumbi	8	2	Yes	2015-10-31	Tertiary hospital
Violence/ terrorism	Western	Karongi	0	3	No	2015-12-24	-
RTA	Northern	Gicumbi	1	11	Yes	2016-01-18	District hospital
RTA	Southern	Huye	4	4	Yes	2016-04-22	Tertiary hospital
Natural hazard	Southern, Northern, Western	Gakenke, Muhanga, Rubavu, Ngororero	72	26	No	2016-05-07	-
RTA	Kigali	Kicukiro	7	9	Yes	2016-06-10	Tertiary hospital
RTA	Western	Rubavu	1	27	Yes	2016-06-13	District hospital
RTA	Kigali	Nyarugenge	4	9	Yes	2016-06-31	Tertiary hospital

RTA	Eastern	Nyagatare	-	-	Yes	2016-09-19	District hospital
Natural hazard	Western	Rusizi	1	20	Yes	2016-09-23	District hospital
Violence/ terrorism	Eastern	Gatsibo	1	3	Yes	2016-10-02	District hospital
RTA	Kigali	Kicukiro	3	21	Yes	2016-10-11	Tertiary hospital
RTA	Kigali	Gasabo	4	-	No	2017-04-18	-
Natural hazard	Northern, Southern, Eastern, Western	Gicumbi, Huye, Bugesera, Kirehe, Ngoma, Nyabihu, Nyamasheke, Rubavu, Rusizi	3	24	No	2017-10-05	-
Natural hazard	Western	Rubavu	0	3	No	2018-03-03	-
Natural hazard	Southern	Nyaruguru	16	140	No	2018-03-10	-
Natural hazard	Northern, Kigali, Eastern	Gatsibo, other (not specified)	18	-	No	2018-04-23	-
Natural hazard	Northern, Western	Karongi, Rubavu	18	12	No	2018-05-06	-
Natural hazard	Eastern	Rwamagana	14	-	No	2019-01-22	-
Natural hazard	Western	Karongi	15	-	No	2019-05-19	-
RTA	Western	Karongi	8	17	Yes	2019-06-16	Tertiary hospital, Provincial hospital
Violence/ terrorism	Northern	Musanze	8	18	No	2019-10-05	-
Natural hazard	Kigali, Eastern	Gatsibo, Gasabo, Kicukiro, Nyarugenge	19	8	No	2020-02-02	-
RTA	Southern	Kamonyi	7	8	No	2020-02-13	-
Natural hazard	Southern, Northern, Western	Gakenke, Nyabihu, Muhanga	65	-	No	2020-05-07	-

RTA = Road traffic accidents.

Appendix 5. Proposed data collection form for systematic media reviews.

- **Article title:** __________
- **Who published article?** (newspaper name) __________
- **Language of article:** English / Other (adapt to relevant context)
- **Date of publication (YYMMDD):** __________
- **Date of trauma event (YYMMDD):** __________
- **Geographical location of the event:** adapt to relevant context
- **What was the mechanism of trauma?** Road traffic accident / natural hazards / Act of violence/terrorism / other
    - If “road traffic accident”:
        - **What type of vehicles/persons were involved in the road traffic accident (check all that apply)?** Bus, car or truck / motorcycle / bicycle / pedestrians / other
        - **What happened in the accident?** Collision / derailment (slid off the road) / Other
    - If “natural hazard”:
        - **What type of natural hazard was it?** Landslide or flooding / earthquake / storm, hurricane or cyclone / other
    - If “Act of Violence/ terrorism”:
        - **What type of act of Violence/ terrorism-related mechanismwas it?** Machete or knife / gun, rifle or firearm / explosion or detonation / other
- **Number of persons injured (not dead; including mild and critical injuries):** __________
- **Number of persons critically injured (if specified):** ___________
- **Number of on-site deaths:** __________
- **To what extent was demographic data (names, age, gender, nationality) provided**? None / data for some of the deceased only / data for all the deceased only / data for some of the victims (injured and deceased) / data for all victims (injured and deceased)
- **Demographic data provided (check all that apply):** names of victims / gender / age / age-group (e.g. “children”, “students” or “elderly”) / none / other
- **Was there information on to which health facilities the victims were taken?** Yes / no / partially
- **To what level of health facility were the victim(s) taken (check all that apply)?** Health center / district hospital / provincial hospital / tertiary hospital
- **Name of hospital patients were taken to:** __________
- **Any other relevant information shared:** __________
