# Supplementary material for: Systematic media review: A novel method to assess mass-trauma epidemiology in absence of databases—A pilot-study in Rwanda
Source: PLoS One. 2021 Oct 13;16(10):e0258446. doi: 10.1371/journal.pone.0258446 (PMC8513851; doi:10.1371/journal.pone.0258446)
Supplement: Attachment — Submitted filename: Response to reviewers_final.docx [file pone.0258446.s009.docx]

Dear Reviewers,

We want to thank you for taking your time to review our text in-depth and for the valid and constructive feedback provided. We have addressed your comments below and made changes accordingly, which we truly believe will make this paper better. If anything is unclear, please do not hesitate to reach out to us for further questions or clarification.

Best regards,

Lotta Velin, M.D.

On behalf of the study team

1. Please ensure that your manuscript meets PLOS ONE's style requirements, including those for file naming. The PLOS ONE style templates can be found at

<https://journals.plos.org/plosone/s/file?id=wjVg/PLOSOne_formatting_sample_main_body.pdf> and

<https://journals.plos.org/plosone/s/file?id=ba62/PLOSOne_formatting_sample_title_authors_affiliations.pdf>

This has been addressed.

2. We note that Figure 2 in your submission contain map images which may be copyrighted. All PLOS content is published under the Creative Commons Attribution License (CC BY 4.0), which means that the manuscript, images, and Supporting Information files will be freely available online, and any third party is permitted to access, download, copy, distribute, and use these materials in any way, even commercially, with proper attribution. For these reasons, we cannot publish previously copyrighted maps or satellite images created using proprietary data, such as Google software (Google Maps, Street View, and Earth). For more information, see our copyright guidelines: <http://journals.plos.org/plosone/s/licenses-and-copyright>.

We require you to either (1) present written permission from the copyright holder to publish these figures specifically under the CC BY 4.0 license, or (2) remove the figures from your submission:

a. You may seek permission from the original copyright holder of Figure 2 to publish the content specifically under the CC BY 4.0 license.

We recommend that you contact the original copyright holder with the Content Permission Form (<http://journals.plos.org/plosone/s/file?id=7c09/content-permission-form.pdf>) and the following text:

“I request permission for the open-access journal PLOS ONE to publish XXX under the Creative Commons Attribution License (CCAL) CC BY 4.0 (<http://creativecommons.org/licenses/by/4.0/>). Please be aware that this license allows unrestricted use and distribution, even commercially, by third parties. Please reply and provide explicit written permission to publish XXX under a CC BY license and complete the attached form.”

Please upload the completed Content Permission Form or other proof of granted permissions as an "Other" file with your submission.

In the figure caption of the copyrighted figure, please include the following text: “Reprinted from [ref] under a CC BY license, with permission from [name of publisher], original copyright [original copyright year].”

b. If you are unable to obtain permission from the original copyright holder to publish these figures under the CC BY 4.0 license or if the copyright holder’s requirements are incompatible with the CC BY 4.0 license, please either i) remove the figure or ii) supply a replacement figure that complies with the CC BY 4.0 license. Please check copyright information on all replacement figures and update the figure caption with source information. If applicable, please specify in the figure caption text when a figure is similar but not identical to the original image and is therefore for illustrative purposes only.

The following resources for replacing copyrighted map figures may be helpful:

USGS National Map Viewer (public domain): <http://viewer.nationalmap.gov/viewer/>

The Gateway to Astronaut Photography of Earth (public domain): <http://eol.jsc.nasa.gov/sseop/clickmap/>

Maps at the CIA (public domain): <https://www.cia.gov/library/publications/the-world-factbook/index.html> and <https://www.cia.gov/library/publications/cia-maps-publications/index.html>

NASA Earth Observatory (public domain): <http://earthobservatory.nasa.gov/>

Landsat: <http://landsat.visibleearth.nasa.gov/>

USGS EROS (Earth Resources Observatory and Science (EROS) Center) (public domain): [http://eros.usgs.gov/#](http://eros.usgs.gov/)

Natural Earth (public domain): <http://www.naturalearthdata.com/>

According to the print right terms from Microsoft, we may use the screenshot of our map if we have an acknowledgement statement of this copyright. We have therefore added the following statement as an acknowledgement: “The map in Figure 2 is a Microsoft product screen shot reprinted with permission from Microsoft Corporation.”

3. Please review your reference list to ensure that it is complete and correct. If you have cited papers that have been retracted, please include the rationale for doing so in the manuscript text, or remove these references and replace them with relevant current references. Any changes to the reference list should be mentioned in the rebuttal letter that accompanies your revised manuscript. If you need to cite a retracted article, indicate the article’s retracted status in the References list and also include a citation and full reference for the retraction notice.

No changes have been made to the reference list.

[Note: HTML markup is below. Please do not edit.]

Reviewers' comments:

Reviewer's Responses to Questions

**Comments to the Author**

Review Comments to the Author

Please use the space provided to explain your answers to the questions above. You may also include additional comments for the author, including concerns about dual publication, research ethics, or publication ethics. (Please upload your review as an attachment if it exceeds 20,000 characters)

Reviewer #1: Comment PLOS One - PONE-D-21-19748
Title – Systematic media review: a novel method to assess mass-trauma epidemiology in absence of databases - a pilot-study in Rwanda

Abstract – Structured and well written

Background –
Detailed information on statement of problem as well as rational for the study clearly presented.

Methods
Well described in details

Result
Well written in details with relevant tables and figures
Page 13, line 168 – 170: Is this multiple response? The different types of transport / vehicle added together is more than 100%. This could not be verified on any of the tables

On Page 13, there is only the references, but if this question refers to the types of vehicles involved in road-traffic accidents detailed on page 7, the answer is “Yes”: all vehicles involved in the accident were selected in this multiple-answer question, for example if the accident involved a bicycle and a car, these two “boxes” were both checked.

Discussion
Well discussed with study limitations provided.

Conclusion –
Clearly written with appropriate recommendation.

Reviewer #2: Systematic media review a novel method to assess mass-trauma epidemiology in absence of database-a pilot study in Rwanda
Much emphasis is given to the problem of MCI in all over the world than the current situation in the local setting about recording of the MCI. To consider the novelty of the proposed system, we need to know the current situation of recording/registration of MCI in Rwanda.

The novelty of the method is less emphasized in the whole presentation of the article. Injury registry has been implemented [lines 92-93, page 4] so where is the novelty of your method? Do you mean this approach will provide information broader in scope as previous data were limited to certain types of disasters [line 93]?

It seems that nothing is special here in Rwanda to implement the current study with novel approach. Please first provide the big picture of the problem i.e., the legacy system that the novel approach could improve.

Answer: Thank you for your questions!

The implementation of a WHO trauma registry at the four selected referral hospitals (CHUK, CHUB, Rwanda Military Hospital and Ruhengeli Referral Hospital) was started in August 2019, with the facilitation from an American grant that covered the necessary pre-implementation logistics and trainings for site leaders, data collectors and data managers. By August 2020, the registry had made about 5000 entries (trauma visits) at the selected hospitals. However, the implementation of the WHO trauma registry only covered 4 tertiary hospitals in urban settings, which limit the information on those patients who reached care at referral hospitals, but not those managed at the many peripheral healthcare facilities, although our media review shows that many mass-casualty incidents occur in and are initially or completely managed in rural areas.

Another important consideration is that in low-income countries, mass-trauma events are common, but as far as we know, no in-hospital registry distinguishes between singles injuries and injuries occurring in connection with a mass-trauma event (as explained on page 3-4, lines 76-81).

Furthermore, although trauma registries are doubtless useful tools to get a picture of epidemiological trends, they collect solely in-hospital data. In countries, as Rwanda, where incidence of trauma is high and access to care limited, the burden of trauma in patients who did not reach care is still unknown. The lack of this information hinders from developing appropriate strategies for preventing injuries, optimize access to necessary care and increase the capacity to manage this kind of injury. Therefore, with the new approach of a media-based review we aimed to assess the epidemiology of mass-trauma events in the entire country, whether they sought care or not.

Finally, maintaining a registry is expensive and time-consuming, which limits their use in countries with limited resources. The strengths and novelty of this method lie in the facts that no external grants nor extensive manpower (in terms of ensuring strict adherence to an implemented registry) is needed to assess the mass-trauma epidemiology and so its usefulness is likely to be transferrable to other countries, regardless of available registries or funding. For these reasons, we think that media-review is a methodology that can be used in other countries as well, independently from their resource availability and including countries that lack any type of injury registry. We have tried to better highlight these points and the importance of this novel methodology in our paper on page 11, lines 297-308.

We would also like to highlight Rwanda’s suitability for piloting a novel project like this. Rwanda has a strong tradition of innovation and leading new global health interventions which later inspire similar actions in other countries. This may be due to Rwanda’s expansive public health system and strong political support for health system strengthening and innovation in healthcare.

• The rationale presented [lines 96-97, page 4] to estimate the burden of MCI in limited resource setting is important. Such report on descriptive type of epidemiology [Person, Place, and Time] is the main component expected from the analysis. The trend in the traumatic injury [Time] is reported [Fig. 1], the geographic distribution [Place] where all the incidents happened during the study period is also well presented [Fig. 2]. However, it seems that the current analysis missed a big component of the demographic structure [Person] of the traumatic injury report to estimate the burden of MCI. Without personal identifier, the age distribution and gender mix of MCI should have been reported to estimate the burden. I think none of the search strategy could not capture such information [Appendix 1]. Age and gender should be the component as keyword in your search strategy to have the demographic distribution. Since such keywords were not used your result could not capture this information and it was reported as if age and gender were not reported less. Demographic data were reported in 37.4% [lines 156-158, page 6], but such inconsistency is a result of the search strategy.

Thank you for this comment! The primary aim of our study was to investigate whether we could assess the epidemiology (incidence and category) of mass-trauma in Rwanda at all with a media-review. We designed therefore a broad search strategy to identify all mass-trauma events that occurred in a selected time period. If we had included “age” and “gender” as words in our search strategy, we would likely have gotten fewer hits, and it would have excluded mass-trauma events that lacked this more in-depth data.

Methods
• The working definition given to MCI is good, but it focused on the frequency of event. What parameters you considered to arrive such arbitrary number should be explained [e.g. hospitals on average in Rwanda can manage at the same time].

We agree that this definition is arbitrary and further research is likely needed to define a more evidence-based threshold for what should be considered an MCI in Rwanda, but this was beyond the scope of this study. The threshold probably varies in different parts of Rwanda, and may also change over time, and therefore, we decided to adopt a pragmatic definition based on the opinion of our Rwandese co-authors, who are expert of the Rwandan trauma system. We also used a study of trauma capacity in Tanzania by Koka et al (as described in our paper, page 12, line 317-318) as basis for what would be realistic in a Rwandan setting. Finally, we adopted a relatively low threshold (n=3) to capture all potential MCIs. We elaborate on this limitation on page 12, lines 323-330.

Further, your definition should include the nature of injuries. Does your definition of MCI work for all-natural calamities and human caused injury incidents contrary to the definition you mentioned [lines 59-60, page 3]?

We have added the word “traumatic” (injuries) in the definition to clarify the scope (page 5, line 118). We prefer not to specify the nature of injuries, such as natural calamities or human caused, in the search strategy because we wanted to capture all kind of multi-trauma across different contexts, to reduce the risk of investigator bias in our search.

• Is death included in your definition of MCI and in the reported traumatic injury? Your result includes death pattern as MCI [line 138-139, page 6]

Thank you for highlighting unclearness in our definition. No, the definition of MCI is “events causing three or more traumatic injuries” without the inclusion of deaths. However, we think that the number of deaths occurred during a MCI is an important marker for MCI severity as clarified on page 6, lines 148-149, which is why we decided to extract and analyse this data as a measurement of MCI epidemiology.

• Please add a few detailed descriptions to the features of the database you used (NexisUni).

Details about NexisUni have been added on page 2 lines 38-39, and it is further described on page 5, lines 122-123.

• For traumatic injury happened in neighboring/adjacent districts outside Rwanda, for example, in a district in Tanzania, but cases were referred to health facilities in one of the districts in Rwanda making the healthcare system overburdened. How do you verify such incidents to include/not in the analysis?

This methodology is intended to provide an overview of mass-trauma epidemiology in a single country. In this paper, the methodology is piloted in Rwanda and so the aim is to detect mass-trauma cases occurring in Rwanda. It is true that theoretically an MCI could occur in a neighboring country with some of the victims being transferred to Rwanda, however it was considered a very rare occurrence by our Rwandese co-authors, and hence a negligible proportion of the mass-trauma burden. However, to verify this hypothesis, it would be beneficial to conduct similar studies in the neighboring countries to assess if any MCIs have led to patient transfers to Rwanda.

• Your search strategy indicates the input data sources used are electronic media. If the MCI happened and document in a non-electronic media, for example, paper-based news report, how such traumatic injury reports were managed not to miss incidents in your analysis?

Most low- and middle-income countries have limited paper-based news reports, but extensive electronic reporting, as access to phones/smartphones is widespread globally. In Rwanda, electronic media are far more common than non-electronic media, and so it was agreed in our team, including the perspectives from our Rwandan collaborators, that it would be highly unlikely that a mass-trauma event would be described in paper-based news but not mentioned in electronic media.

• In the data analysis subsection, it is reported that Fisher’s exact test is used, but the result presentation did not show any findings related to such analysis.

Fisher’s exact test was used to detect the statistically significant difference in types of mass-trauma events in different regions as presented on page page 8, lines 200-202.

Results
• Any mechanism applied to verify for cases not to be counted twice? In the place where the incident happened and if they referred into the next higher level in the health care system and got news attention there will be a possibility of multiple count of cases. [lines 154-156, page 6].

As described in the section ”Data analysis” on page 6, lines 141-147: *“in cases where different articles described an MCI with the same trauma mechanism, province, and date (+/- one day), this was considered as the same event, unless there was information that indicated these were distinct MCIs.”*

Furthermore, as a follow-up to this study, we are planning a multicentre study to validate the method of “media-review” and that will allow to distinguish unique cases among “double-counting”. In this project, we will collect retrospective data from hospital records on trauma patients admitted at the four largest hospitals in Rwanda and analyze this in relation to the media review findings.

• Figure 2 needs revisions on the colour resolution applied. The legend showing the colour depth ranges from 0-15 and 0 should be white as there is no value. But that is not the case in the figure. It is good to add values in the picture for each region together with the label name for each district.

To distinguish the color legend from the white background, 0 is marked as very light blue. We have however added the values for each region together with the label name as suggested and we hope that this adds clarity.

Discussion
• Mortality in mass-trauma events did not improve during the study period [lines 220-221, page 9]. Where this result and conclusion come from as the definition did not include death rather the number of injuries was a prioritized concept?

According to data on deaths by MCI founded by the media search, the number of annual deaths due to MCIs was not reduced during the study period. However, we agree that the wording “mortality” is not correct and so we have adjusted this to “number of deaths”, page 9, line 230 and page 13, lines 357-358.

• Please reflect more on the novelty of the method together with its possible benefits and advantages the case at hand over the already existed system.

This has been developed on page 11, lines 300-310, which reads:

*We believe that this methodology has a great potential of filling a large data gap on mass-trauma epidemiology in settings lacking systematic data collection. With the level of detailed data appearing to be lower than data collection through clinical trauma registries, it would be ideal if this method could be seen as a complement to clinical data collection to ensure an in-depth understanding of the implications on the wider health system. Furthermore, in countries such as Rwanda, where incidence of trauma is high and access to care limited, the burden of trauma in patients who did not reach care is still unknown – a knowledge gap which may be addressed through the pre-hospital data collection in a systematic media review. However, with many LMICs lacking resources to develop and maintain trauma registries, the systematic media review could be a cost-effective and easy alternative, with the flexible methodology likely generalizable to different contexts although this should be assessed in future studies.*

• In one of the limitations of the study, the current study finding is compared with respective ministry reports. Such analysis is not evidenced in the result section and no attempt was made to verify/validate the current study estimate against state report. Why it appeared out of the blue in the limitation?

To compare our findings with ministry report was not the aim of our study and not a part of the data collected to answer to our aim. However, we wanted to use the ministry reports in the discussion session since we considered it relevant literature which could be used to bring more context to our findings. We agree that this may add to confusion and have therefore removed the data from the ministry report in the limitations, and just reference the reports (as previously) in the introduction.

• it is suggested that MCI protocols in Rwanda should put an emphasis on rural areas. But for international readers to take this as a key message from the current study, please show the urban rural disparity in terms of MCI in the geographic distribution analysis.

Rwanda is a small but densely populated country, without a clear distinction of what would generally be considered as a rural area and what would be considered as an urban area, making such analysis difficult to achieve. Furthermore, most mass-casualty incidents were only specified at a district level, which contain both urban and rural areas. For these reasons, we have compared Kigali (the capital province) with the other provinces, as a proxy for urban vs rural events – page 7--8, lines 187-192.
